# Evaluation of CO_2_ and H_2_O Adsorption on a Porous Polymer Using DFT and In Situ DRIFT Spectroscopy

**DOI:** 10.1021/acs.jpcb.2c03912

**Published:** 2022-09-28

**Authors:** Giulia E. M. Schukraft, Ioanna Itskou, Robert T. Woodward, Bart Van Der Linden, Camille Petit, Atsushi Urakawa

**Affiliations:** † Barrer Centre, Department of Chemical Engineering, South Kensington Campus, Imperial College London, London SW7 2AZ, U.K.; ‡ Institute of Materials Chemistry and Research, Faculty of Chemistry, University of Vienna, Währinger Straße 42, 1090 Vienna, Austria; § Catalysis Engineering, Department of Chemical Engineering, Delft University of Technology, Van der Maasweg 9, 2629 HZ Delft, The Netherlands

## Abstract

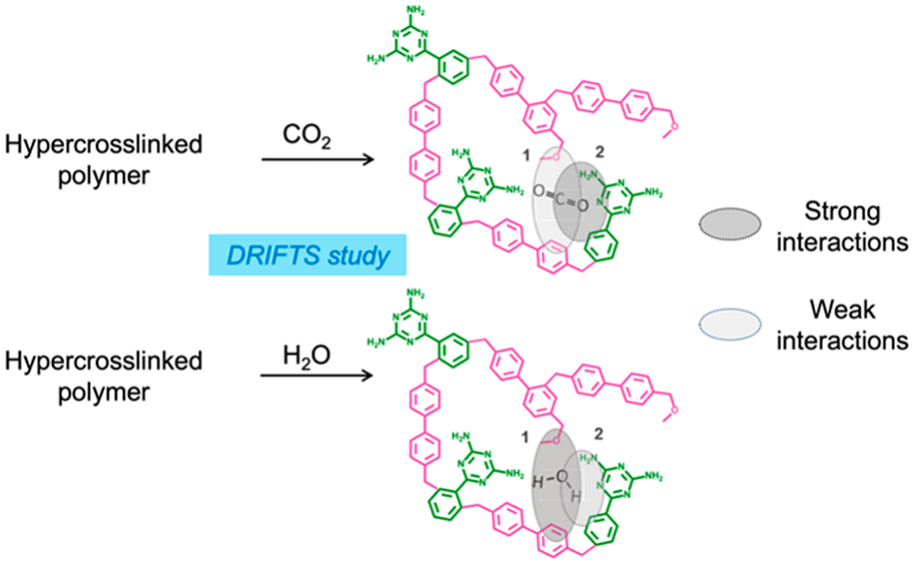

Numerous hyper-cross-linked polymers (HCPs) have been developed as CO_2_ adsorbents and photocatalysts. Yet, little is known of the CO_2_ and H_2_O adsorption mechanisms on amorphous porous polymers. Gaining a better understanding of these mechanisms and determining the adsorption sites are key to the rational design of improved adsorbents and photocatalysts. Herein, we present a unique approach that combines density functional theory (DFT), in situ diffuse reflectance infrared Fourier transform spectroscopy (DRIFTS), and multivariate spectral analysis to investigate CO_2_ and H_2_O adsorption sites on a triazine–biphenyl HCP. We found that CO_2_ and H_2_O adsorb on the same HCP sites albeit with different adsorption strengths. The primary amines of the triazines were identified as favoring strong CO_2_ binding interactions. Given the potential use of HCPs for CO_2_ photoreduction, we also investigated CO_2_ and H_2_O adsorption under transient light irradiation. Under irradiation, we observed partial CO_2_ and H_2_O desorption and a redistribution of interactions between the H_2_O and CO_2_ molecules that remain adsorbed at HCP adsorption sites.

## 1 Introduction

Hyper-cross-linked polymers (HCPs) are a class of amorphous porous material built via the dense cross-linking of organic building blocks.^1^ The ability to “knit” together an extensive set of aromatic compounds without the need for specific polymerizable groups enables a wide structural and chemical diversity. HCPs exhibit a permanent porosity and high surface area, and their synthesis is straightforward, requiring only common Lewis acid catalysts to initiate polymerization, such as Fe(III) chloride and Al(III) chloride, or organic Brønsted acids.^2,3^ This simplicity and potential for scale-up explain the scientific interest around these materials. In fact, some HCPs are already commercially available (e.g., ion-exchange polymeric resins) while others are being developed and tailored for different applications related to gas separation/storage,^4−6^ liquid phase adsorption,^7,8^ and catalytic organic transformations.^9−12^ In particular, HCPs have recently been investigated for CO_2_ capture^6,7,13−18^ and CO_2_ photoconversion to value-added chemicals.^19,20^ Martin et al. reported a biphenyl–xylene HCP with CO_2_ uptake capacity of 1.7 and 13.4 mmol g^–1^ under atmospheric pressure and 30 bar, respectively, exceeding that of commercial adsorbents like zeolite 13X and activated carbons BPL and Norit R under similar conditions.^13^ Su et al. studied an amine-functionalized HCP that displayed selective CO_2_ uptake over N_2_, a feature they attributed to the high density of primary amines and the relatively small pore size of the material.^7^ Beyond CO_2_ capture, the use of HCPs as photocatalysts for CO_2_ reduction is also emerging. Wang et al. reported a HCP–TiO_2_–graphene composite for photocatalytic CO_2_ reduction, with the HCP component facilitating CO_2_ adsorption and diffusion.^20^ However, the ability of HCPs alone to catalyze solar fuel production has remained unknown until recently, when we have demonstrated their activity for CO_2_ photoreduction using water vapor as sacrificial agent.^19^ A triazine–biphenyl-based HCP synthesized via a Friedel–Crafts alkylation reaction has showed a CO_2_ photoconversion rate comparable to that of TiO_2_ P25.

In both CO_2_ capture and CO_2_ catalytic conversion, CO_2_ and H_2_O adsorptions represent critical steps that one could a priori control to optimize gas diffusion, adsorption, CO_2_ reaction/activation, and product selectivity. Such control relies on understanding adsorption mechanisms and thermodynamics. As demonstrated on other materials such as zeolites,^14,21^ nonporous linear polymers,^22^ alumina,^23,24^ TiO_2_,^25,26^ and other metal oxides,^27^ in situ infrared spectroscopy can provide useful insights into adsorption and photoconversion mechanisms. For example, Wilfrong et al. investigated CO_2_ adsorption and mass transfer across a tetraethylenepentamine film using in situ diffuse reflectance infrared Fourier transform spectroscopy (DRIFTS) and in situ attenuated total reflection infrared spectroscopy (ATR-IRS) measurements.^22^ In photocatalysis applications, in situ IR techniques can also provide insights into the CO_2_ adsorption mechanisms and the nature of the active sites. Mino et al. studied CO_2_ adsorption on different facets of TiO_2_ anatase.^25^ By combining DFT and in situ ATR-IRS, they revealed that CO_2_ weakly interacted with the (101) surface and adsorbed without bending, while on the (001) surface, CO_2_ adsorbed in the form of carbonates. Studying CO_2_ adsorption under transient irradiation using in situ FTIR spectroscopy can also lead to a better understanding of product selectivity. Ordoño et al. explored how Pt and Co cocatalysts impacted the product selectivity of TiO_2_ in CO_2_ photoreduction using H_2_O as sacrificial agent.^26^ Time-resolved in situ DRIFTS helped identify surface formates as active intermediate species when using Pt or Co as cocatalysts.

As these studies show, in situ infrared spectroscopy can provide useful insights into CO_2_ adsorption and photoconversion mechanisms. To date, there is limited understanding of CO_2_ and H_2_O adsorption mechanisms on amorphous porous polymers such as HCPs, which prevents the rational design of optimized HCP adsorbents and photocatalysts for CO_2_ management. Yet, the use of IR to study CO_2_ adsorption mechanisms on HCPs, while attractive, is not without challenges. For instance, the organic nature of HCPs means that many of the relevant IR absorption bands of the polymers and CO_2_ may overlap.

Herein, we present a unique approach that combines DFT, in situ DRIFTS, and multivariate spectral analysis to investigate CO_2_ and H_2_O adsorption on a triazine–biphenyl HCP (being the best performing catalyst in our previous CO_2_ photoreduction study).^19^ The presence of triazine groups enabled us to investigate the role of a nitrogen-rich functional group on CO_2_ and H_2_O adsorption. We first assigned the HCP vibrational bands and then identified the HCP vibrational fingerprints involved in intermolecular interactions with CO_2_ and H_2_O. We found that CO_2_ and H_2_O adsorb at the same HCP sites albeit with different adsorption strengths, which led to different desorption patterns upon either heating or light irradiation. Finally, our DRIFTS analysis enabled us to confirm the network formation mechanism of the triazine–biphenyl HCP.

## 2 Methodology

### 2.1 Experimental Section

All reagents used in this study were of analytical grade and used without further purification. 2,4-Diamino-6-phenyl-1,3,5-traizine (CAS # 91-76-6), triflic acid (CAS # 1493-13-6), 1,2-dichloroethane (CAS # 107-06-2), and ethanol (CAS # 64-17-5) were purchased from Sigma-Aldrich, while 4,4-bis(methoxymethyl)biphenyl (CAS # 3753-18-2) was purchased from TCI Chemicals. ^12^CO_2_ (grade 4.0, >99.99%), N_2_ (grade 5.0, >99.999%), and He (grade 5.0, >99.999%) were purchased from Linde Gas Benelux. ^13^CO_2_ (99.0 at. % ^13^C) was purchased from Aldrich.

#### 2.1.1 Hyper-Cross-Linked Polymer Synthesis

##### Triazine–Biphenyl HCP

1.2 g of 4,4-bis(methoxymethyl)biphenyl and 0.46 g of 2,4-diamino-6-phenyl-1,3,5-traizine were dissolved in 18 mL of 1,2-dichloroethane. 0.98 mL of triflic acid was then added dropwise while stirring. The mixture was kept under stirring at 90 °C for an additional 18 h. The resulting polymer was recovered and washed via Soxhlet extraction with 150 mL of ethanol for 24 h, followed by 150 mL of 1,2-dichloroethane for 48 h. The polymer was then dried under vacuum at 150 °C and finely ground. In this study, we refer to 4,4-bis(methoxymethyl)biphenyl as a biphenyl monomer and 2,4-diamino-6-phenyl-1,3,5-traizine as a triazine monomer.

#### 2.1.2 Triazine Solution Preparation

15 mg of triazine monomer was dissolved in 1 mL of THF to form an 80 nM triazine solution. The solution was then sonicated for 30 s at 25 °C.

#### 2.1.3 Hyper-Cross-Linked Polymer Characterization

##### Textural, Structural, and Morphological Properties

Powder X-ray diffraction (PXRD) measurements were performed at room temperature on a Bruker 2D PHASE diffractometer operating at 30 kV and 10 mA with monochromatized Cu Kα radiation (λ = 0.15418 nm). The morphology of the sample was studied using a scanning electron microscope (SEM) (Leo Gemini 1525, Zeiss) in secondary electron mode (InLens detector) at 5 kV. The sample was ground and deposited on carbon tape. To reduce charging, the sample was coated with 17 nm of chromium. Nitrogen sorption isotherms were collected using a Micromeritics 3Flex volumetric sorption analyzer at −196 °C. Prior to analysis, all samples were degassed overnight at 150 °C at around 0.2 mbar. An additional in situ degas step of 4 h was performed at 0.003 mbar and 150 °C. The surface area and the micropore volume (*V*_MICRO_) were calculated using the Brunauer–Emmett–Teller (BET) and DeBoer *t*-plot theory, respectively.^28,29^ The total pore volume (*V*_TOT_) was calculated at *P*/*P*_0_ = 0.99. The pore size distribution was derived from a DFT carbon split shape model built into the Micromeritics software.

##### Chemical Properties

Thermogravimetric analyses (TGA) were performed using a Netzsch TG209 F1 Libra thermogravimetric analyzer. Around 15 mg of sample was heated to 900 °C under N_2_ or air with a temperature ramp of 10 °C min^–1^. An initial isothermal step of 1 h was performed at 120 °C under a N_2_ or air atmosphere to remove any adsorbates. A total flow rate of 100 mL min^–1^ was used. Solid-state ^13^C NMR analyses were conducted on a Bruker 600 MHz AVANCE III HD spectrometer. Around 20 mg of sample was packed into a 3.2 mm zirconia rotor. The spinning was set at 14 kHz with a relaxation delay of 4 s. A total of 2028 scans were collected. Inductively coupled plasma optical emission spectroscopy (ICP-EOS) measurements were then conducted to determine the Fe content. These analyses were performed by MEDAC Ltd. 25 mg of sample was digested using sulfuric and perchloric acid. Elemental analysis was performed on a Eurovector EA 3000 CHNS-O elemental analyzer. A micro-Sartorius ME 5 OCE balance was used to weigh around 0.5 mg of sample. To ensure accuracy, duplicate tests were performed. Combustion and reduction were performed at 1000 °C (1480 °C for O analysis) and 750 °C, respectively. High-purity helium (>99.999%) was used as a carrier gas. X-ray photoelectron spectroscopy (XPS) measurements were performed on a Thermo Scientific K-Alpha+ X-ray photoelectron spectrometer. The instrument is equipped with a MXR3 Al Kα monochromated X-ray source (*h*ν = 1486.6 eV). The X-ray gun power was set to 72 W (6 mA and 12 kV). Prior to analysis, the triazine–biphenyl HCP was ground and deposited on the XPS holder using a conductive carbon tape. Data analysis was performed using the Thermo Avantage software. All the samples were referenced against the C–C peak of adventitious carbon in the C 1s spectrum at a binding energy of 284.8 eV.

##### Optoelectronics Properties

Diffuse reflectance ultraviolet–visible (DR-UV/vis) spectra were obtained using a Shimadzu UV-2600 spectrometer equipped with an integrating sphere. The absorption spectra were derived using the Kubelka–Munk function.^30,31^

##### Gas Adsorption Properties

CO_2_ and water vapor sorption isotherms were collected at 25 and 50 °C using a Micromeritics 3Flex volumetric gas sorption analyzer. Sample were degassed overnight at 150 °C at around 0.2 mbar and again in situ at 150 °C and 0.003 mbar for 4 h. For water vapor sorption measurements, the sorption analyzer was equipped with a liquid container filled up with Milli-Q water with a resistance of >18.2 μohm. Prior to analysis, the Milli-Q water was purified through four freeze–pump–thaw cycles. For CO_2_ sorption measurements, a research grade (99.999%) CO_2_ gas cylinder was used.

##### Infrared Spectroscopy Characterization

ATR-FTIR measurements were performed on a NEXUS from Thermo Nicolet instrument equipped with a liquid N_2_ cooled MCT detector. A total of 220 scans were collected. In situ DRIFTS measurements were performed on a Nicolet 8700 Thermo Scientific FTIR instrument equipped with a liquid N_2_ cooled MCT detector. A Praying Mantis optical accessory from Harrick was used. The optical accessory included a high-temperature and low-pressure Harrick cell and a series of optical mirrors to collect the refracted IR beam. The in situ cell was made of three windows: two IR transparent windows made of BaF_2_ and one window made of fused silica for UV–vis irradiation. A thermocouple was used to control the temperature inside the cell. Prior to the analysis, a few milligrams of the sample was loaded inside the cell and heated at 150 °C for 18 h under He flow (30 mL min^–1^). When needed, the temperature was then lowered to 50 or 100 °C. CO_2_ adsorption measurements were performed by alternatively passing CO_2_ and He through the setup. At first, the atmosphere was switched to a CO_2_/He flow of 6.6 mL min^–1^ with a 2:1 ratio for 50 min and then switched to a He flow of 30 mL min^–1^ for another 50 min. Two CO_2_/He–He cycles were performed. Prior to passing any CO_2_ through the DRIFTS cell, a DRIFT spectrum of the HCP was acquired under a He atmosphere and used as background spectrum for the subsequent experiments. For H_2_O adsorption measurements, water-saturated He and He were alternately passed through the DRIFTS cell. At first, the atmosphere was switched to a water-saturated He stream of 6.6 mL min^–1^ for 50 min and switched to a He flow of 30 mL min^–1^ for 50 min. Two water-saturated He–He cycles were performed. Prior to passing any water vapor through the DRIFTS cell, a DRIFT spectrum of the HCP was acquired under a He atmosphere and used as background spectrum for the subsequent experiments. For transient irradiation experiments, a SwiftCure PLU-10 lamp equipped with an optical fiber and a high-pressure Hg lamp (250 W) was used. The sample was exposed to a CO_2_/water-saturated He atmosphere (1.5 vol/vol ratio) for 1 h without light irradiation. The UV–vis lamp was then turned on for 3 h, and only afterward transient irradiation was undertaken for an additional 3 h. During transient irradiation, the light was turned on and off every 5 min. For all DRIFTS measurements, scans were collected every 12 s. An optical velocity of 1.8988 cm s^–1^ was used. Spectra were recorded in single beam mode. For isotopic DRIFTS measurements, a similar procedure to that described above was used. The HCP under investigation was exposed to a water-saturated ^13^CO_2_ atmosphere instead of a water-saturated ^12^CO_2_ atmosphere and then irradiated with UV–vis light. We note that swelling and any related change in the refractive index of the HCP upon gas adsorption would affect the intensity of the IR signal but not the band position.

### 2.2 Computational Section

All DFT calculations were performed with B3PW91 functional using Gaussian 09. For the geometry optimization and calculation of infrared spectra of one triazine and one biphenyl molecule, the 6-311G(2d,2p) basis set was used. All calculations were performed in vacuo. For the calculation of the six triazine molecular cluster, an initial geometry optimization was performed using the PM3 semiempirical method followed by the DFT calculation with the 3-21 basis set. The output file was then further optimized using the 6-311G(2d,2p) basis set. To compare the simulated infrared spectra with the experimental ones, an empirically determined scaling factor of 0.973 was used for the triazine molecules, while a scaling factor of 0.970 was used for the biphenyl molecules.

The data obtained by time-resolved DRIFTS were processed by multivariate spectral analysis, specifically multivariate curve resolution (MCR) analysis.^32−34^ This approach allowed us to extract the kinetically pure spectra of the vibrational bands involved in CO_2_ or H_2_O adsorption as well as the corresponding concentration profiles. When performing MCR, the number of components was determined to be 6 and 7 for CO_2_ and H_2_O adsorption measurements, respectively. For transient irradiation measurements, the number of components used was 10 based on principal component spectral analysis, their spectral features, and quality of spectral separation. For all MCR analysis, a pure variable detection method was used as initial estimation. A non-negativity constraint and a convergence criterion of 0.1 were also applied. We note that among the extracted MCR components only the spectrally significant ones are shown in this study.

## 3 Results and Discussion

### 3.1 Hyper-Cross-Linked Polymer Characterization

**Figure 1:**
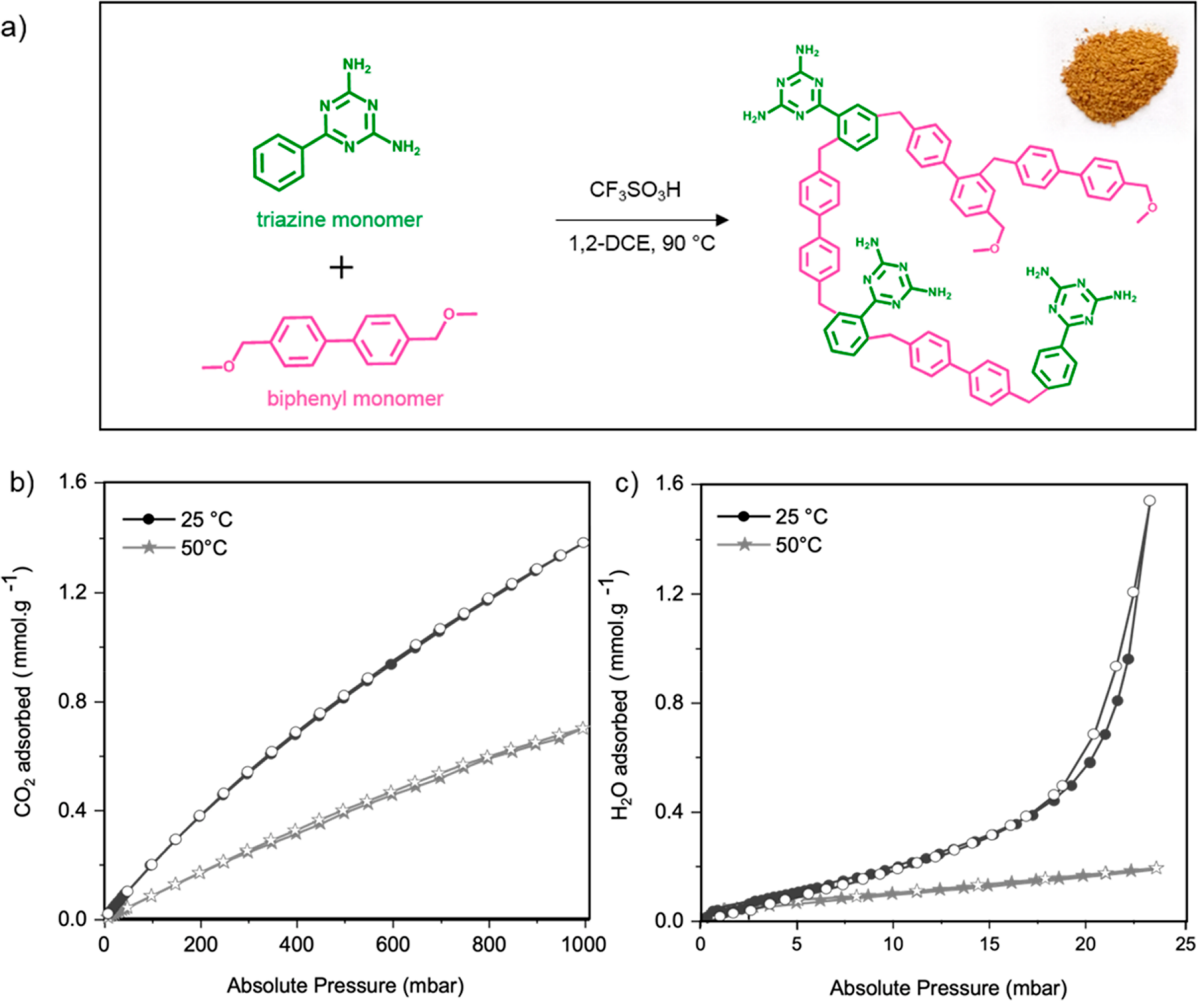
Overview of the chemical features and sorption properties of the triazine–biphenyl HCP sample: (a) synthesis reaction scheme (inset shows a photograph of the polymer); (b) CO_2_ and (c) H_2_O sorption isotherms at 25 and 50 °C of the studied triazine–biphenyl HCP. Filled symbols = adsorption; empty symbols = desorption.

We synthesized a triazine–biphenyl HCP via the cross-linking of 4,4-bis(methoxymethyl)biphenyl and 2,4-diamino-6-phenyl-1,3,5-triazine, subsequently termed biphenyl and triazine monomers, respectively. Triflic acid was used as the polymerization catalyst. Figure 1a illustrates the reaction scheme and the structure of the studied HCP. The photographic and scanning electron images of the polymer reveal a light brown material constituted of agglomerated particles of around 40 nm in diameter (Figure S1).

We confirmed the synthesis of the HCP sample and analyzed its chemical, structural, and optoelectronic features using ssNMR, elemental analysis, X-ray photoelectron spectroscopy, N_2_ sorption at −196 °C, XRD, and UV–vis diffuse reflectance spectroscopy (Figures S1 and S2, Table S1). The HCP sample contains 0.62 at. % N, corresponding to a 1:31 triazine to biphenyl monomer ratio, and 1.1 at. % O, which suggests the presence of methoxy groups arising from partially cross-linked biphenyl molecules. It has a BET surface area of 1247 m^2^ g^–1^ and exhibits a type IV N_2_ sorption isotherm and a multimodal pore size distribution with predominance of micropores and mesopores (Figure S3, Table S2). Powder X-ray diffraction confirmed the amorphous nature of the HCP network (Figure S4). The sample adsorbs light in both the UV and visible regions of the spectrum with an absorption offset at 351 nm (Figure S1d). Finally, we assessed its thermal stability using thermogravimetric analysis and confirmed thermal stability up to 250 °C in both N_2_ and air (Figure S5).

### 3.2 Hyper-Cross-Linked Polymer Sorption Properties

Having evaluated the chemical, structural, and light absorption properties of the triazine–biphenyl HCP, we next investigated the sorption at 25 and 50 °C of CO_2_ and H_2_O—two species present in the CO_2_ capture and CO_2_ photoreduction processes. The former temperature facilitates comparison with published literature while the latter corresponds to the temperature at which we performed CO_2_ photoreduction measurements.

As Figure 1b shows, the HCP sample displays CO_2_ uptake capacities at 1 bar and at 25 and 50 °C of 1.38 and 0.70 mmol g^–1^, respectively. To provide some perspective, zeolite 13X, a benchmark CO_2_ adsorbent, exhibits a CO_2_ uptake capacity of ∼4 mmol g^–1^ at 1 bar and 25 °C.^35^ Compared to other HCPs, the studied HCP displays relatively similar adsorption capacities (Table S3).^7,13^

With regard to H_2_O sorption, at 23 mbar and at 25 and 50 °C, the studied HCP displays a low H_2_O uptake capacity of 1.5 and 0.17 mmol g^–1^, respectively (Figure 1c, to be compared to 16.7 mmol.g^–1^ at 25 °C and 23 mbar for zeolite 13X).^36^ Despite a higher surface area, the HCP studied here adsorbs considerably less H_2_O compared to a previously investigated benzene-based hyper-cross-linked polymer, which displayed a surface of 931 m^2^ g^–1^ and a H_2_O uptake capacity of 9.4 mmol g^–1^ at 25 °C.^19^ The relatively low H_2_O uptake capacities of the triazine-biphenyl HCP likely results from the low oxygen content, i.e., 1.1 at. % O compared to 5.9 at. % O for the benzene-based HCP.

### 3.3 Vibrational Analysis and Band Assignment

Having evaluated the sorption properties of HCP, we next explored how CO_2_ and H_2_O adsorb on the HCP surface using infrared spectroscopy. To identify the HCP vibrational fingerprints involved in intermolecular interactions with CO_2_ and H_2_O, one must assign the HCP’s vibrational bands. In theory, one could use the computed infrared spectra of a single triazine and biphenyl molecule and compare them with the measured infrared spectrum of the HCP. However, because of the complex interactions between monomer molecules after polymerization, the experimental HCP infrared spectrum will differ from the simulated ones. Hence, to achieve a precise band assignment, we must understand how intermolecular interactions impact the infrared spectrum of a triazine molecule and that of a biphenyl molecule, i.e., bands shifts, band broadening, and the appearance of new vibrational bands. A detailed description of our assignment approach and the resulting band assignment can be found in the Supporting Information (Section 2, Tables S4–S6, Figures S6 and S7).

### 3.4 Vibrational Band Analysis during CO_2_ Adsorption

**Figure 2:**
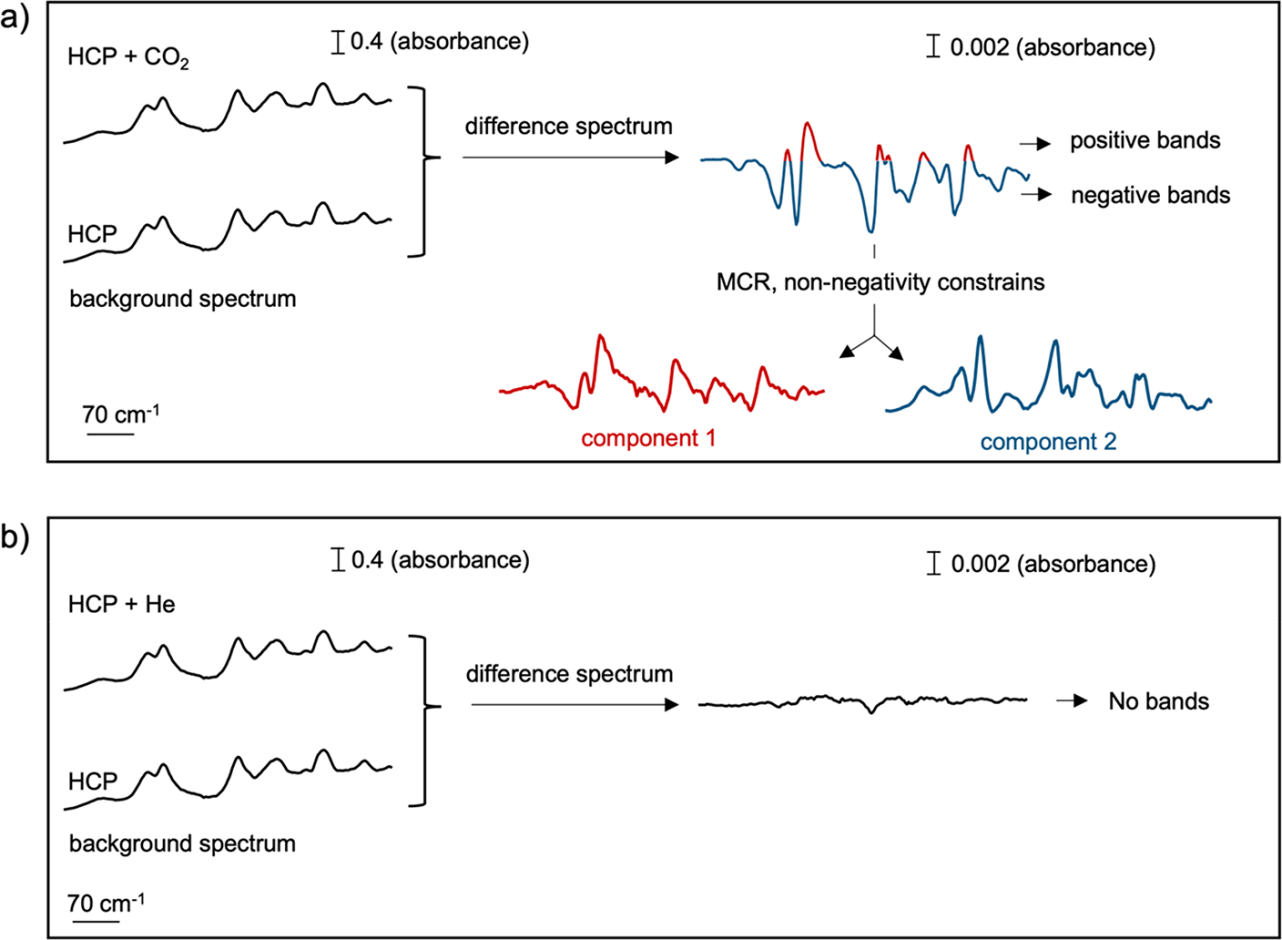
Representation of the spectral components obtained upon processing the difference spectrum using multivariate curve resolution (MCR). (a) The represented difference spectrum corresponds to the average of the difference spectra acquired under a CO_2_ atmosphere at 50 °C. (b) The represented difference spectrum corresponds to the average of the difference spectra acquired under a He atmosphere at 50 °C.

Having assigned the HCP vibrational bands, we next evaluated those involved in CO_2_ adsorption to identify the CO_2_ adsorption sites. To do this, we conducted time-resolved in situ DRIFTS measurements by alternatingly passing CO_2_ and He through the DRIFTS cell. We performed two CO_2_–He cycles for each temperature studied (50, 100, and 150 °C). Prior to passing CO_2_ through the DRIFTS cell, we collected the DRIFT spectrum of the HCP under He and used it as background spectrum. As Figure 2a shows, under CO_2_ at 50 °C, the obtained difference spectra display negative and positive bands, while under He, these spectral features are not visible (Figure 2b).

To understand the origin of these positive and negative bands, one must remember that the analyzed data are obtained by subtracting the background spectrum (i.e., HCP spectrum in the absence of CO_2_) from the HCP spectrum acquired under a CO_2_ atmosphere. If the background spectrum exhibits spectral features that are absent in the spectrum under a CO_2_ atmosphere, negative bands emerge due to the subtraction. Conversely, if the spectrum obtained under a CO_2_ atmosphere exhibits spectral features absent in the background spectrum, positive bands emerge. Importantly in infrared spectroscopy, the concomitance of negative and positive bands in a difference spectrum is often the result of band shifts. As Figure S9 illustrates, when the vibrational band of a material shifts compared to the background spectrum, the difference spectrum displays a negative band at the original band position and a positive band at the new band location. The relative positions of the negative and positive bands in the difference spectrum enable to determine the direction of the band shift. If negative bands precede positives ones (left to right; higher to lower wavenumbers), it indicates that the vibrational bands are shifting toward lower wavenumbers (Figure S9a). Conversely, if positive bands precede negative ones, it indicates that the vibrational bands are shifting toward higher wavenumbers (Figure S9b). In this study, as shown by the difference spectra in Figures 2a and S10, under CO_2_, negative bands precede positive bands, indicating that some of the HCP vibrational bands shifted toward lower wavenumbers. This red-shift results from CO_2_ adsorption on the HCP surface. Yet, the complexity of the obtained difference spectrum prevents a precise identification of the HCP vibrational bands that are red-shifted.

**Figure 3:**
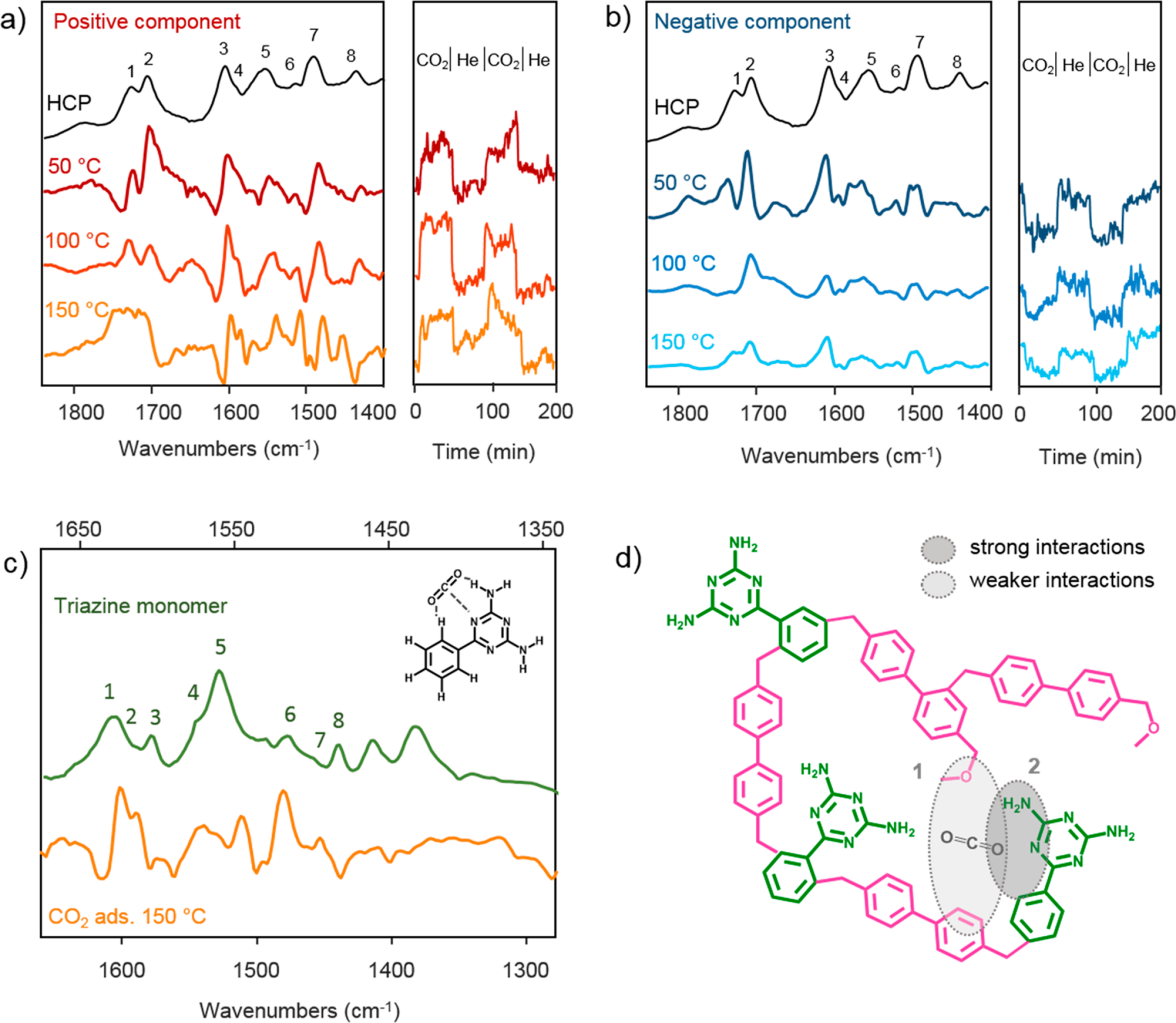
DRIFTS study of CO_2_ adsorption on HCP. Left panels of (a) and (b): comparison between the DRIFT infrared spectrum of the triazine–biphenyl HCP sample with the positive (a) and negative (b) spectral components obtained for CO_2_ adsorption measurements at 50, 100, and 150 °C after performing MCR analysis. The right panels of (a) and (b) represent the component concentration profiles obtained by MCR. (c) Comparison between the ATR infrared spectrum of the triazine monomer with the positive spectral components obtained for CO_2_ adsorption measurements at 150 °C after MCR analysis. (d) Illustration of the CO_2_ adsorption sites in triazine–biphenyl HCP.

To address this challenge, one can process and disentangle the differences in the spectra using multivariate curve resolution (MCR). MCR is a mathematical method that extracts the individual sources of variations present in a signal.^37^ When applied to time-resolved DRIFTS, MCR can extract kinetic behaviors. It identifies the vibrational bands’ kinetics, i.e., the speed at which bands shift or appear or disappear. On the basis of the vibrational bands’ kinetics, MCR separates them into kinetically pure spectral components. Vibrational bands that have identical kinetic behaviors, i.e., (dis)appearing at the same time, are associated with the same component. The relative intensity of those kinetically pure components to the global signal are also determined and are termed a concentration profile. When processing the data using MCR in this study, a non-negativity constraint is applied; i.e., vibrational bands cannot be negative. As Figure 2a illustrates, this constraint converts the negative spectral features of the difference spectrum into positive ones and describes their “negative” intensity by assigning them negative concentrations. After applying the non-negativity constraint, negative and positive spectral features respectively exhibit negative and positive concentration profiles, thereby behaving kinetically differently. Hence, MCR separates them into two different components (Figure 2). The first component represented in red (Figure 3a) corresponds to the positive spectral features of the difference spectra and the second component in blue (Figure 3b) to the negative ones.

As shown by the concentration profiles, at 50 °C, both positive and negative components dynamically appear or disappear in the presence or absence of CO_2_, respectively (Figures 3a and 3b, right panels). This finding corroborates the observation made using unprocessed difference spectra suggesting the presence of both positive and negative bands due to CO_2_ adsorption on the HCP surface. While we cannot rule out that some CO_2_ may still be adsorbed after the desorption step, the intensity of the oscillation in the concentration profiles suggests a reversible process.

After MCR analysis, an exact determination of the HCP vibrational bands red-shifted upon CO_2_ adsorption is now possible. As shown in Figures 3a and 3b, at 50 °C, both negative and positive components display vibrational bands at 1726, 1704, 1605, 1551, and 1490 cm^–1^ (bands 1, 2, 3, 5, and 7 of Figure 3a,b). These bands are the ones shifted upon CO_2_ adsorption on HCP. They correspond to the intermolecular biphenyl C=O bond vibrations, NH_2_ scissoring vibrations coupled with different C–NH_2_ vibrations modes, and N–C=N stretching vibrations of the triazine monomer. Comparing the position of the vibrational bands present in the positive component with the corresponding unshifted HCP vibrational bands, we estimate that upon interaction with CO_2_ the HCP vibrational bands located at 1726, 1704, 1605, 1551, and 1490 cm^–1^ are shifted by −2, −6, −6, −11, and −8 cm^–1^, respectively (Figure S11). To verify that these bands are indeed the ones shifting under a CO_2_ atmosphere, we simulated the difference spectrum obtained when the above-mentioned bands are red-shifted by 8 cm^–1^. As shown in Figure S12, the simulated difference spectrum is almost identical with the one measured, corroborating our hypothesis. The small differences we observe are expected as each vibrational band interacts differently with CO_2_, and therefore not all bands will shift to the same extent, as in our simulation.

Another aspect to study using MCR analysis is the kinetic behavior of the positive and negative vibrational bands present in the difference spectra. As determined by MCR, all the positive vibrational bands belong to the same kinetically pure component and therefore dynamically appear or disappear at the same rate in the presence or absence of CO_2_. A similar reasoning applies to the negative vibrational bands. Following this analysis, the HCP’s vibrational bands located 1726, 1704, 1605, 1551, and 1490 cm^–1^ are all shifting at the same rate under a CO_2_ atmosphere. This observation can only occur if, upon CO_2_ adsorption, CO_2_ molecules interacts with all five vibrational bands at the same time, within the time resolution of our measurements. Thereby, as Figure 3a illustrates, at 50 °C, CO_2_ concurrently interacts with the intermolecular biphenyl C=O bond, the primary amines, and the triazine groups of the triazine monomer. While we anticipated the interactions between CO_2_ and the primary amines and the triazine groups, we did not foresee CO_2_ interaction with the intermolecular biphenyl C=O bond. It is interesting to see that residual oxygen from the incomplete conversion of methoxy groups to cross-links, an a priori undesired feature, plays a role in CO_2_ adsorption.

To investigate the strength of CO_2_ adsorption with the above-mentioned functional groups, we conducted additional CO_2_ adsorption measurements at 100 and 150 °C. At higher temperatures, weakly bound CO_2_ desorbs, and only the spectral features promoted by strongly adsorbed CO_2_ are observed. As Figure 3a shows, when increasing the temperature to 100 and 150 °C, the vibrational bands of the positive component corresponding to the intermolecular biphenyl C=O bond gradually disappear (bands 1 and 2), while the ones corresponding to the triazine monomer remain unchanged (bands 3, 5, and 7). A similar behavior is observed for the negative component: the negative vibrational bands of the intermolecular biphenyl C=O bond disappear faster than those of the triazine monomer. Such observations indicate that CO_2_ adsorbs more strongly to the triazine groups than to the biphenyl sites. At 150 °C, we only observe the vibrational bands corresponding to the triazine monomer (Figure 3c), suggesting that at 150 °C, CO_2_ mainly adsorbs on the triazine monomer. We illustrate how CO_2_ interacts with the triazine component of the HCP in Figure 3d. As the temperature increases from 50 to 150 °C, some of the positive triazine vibrational bands become sharper (Figure 3a) as strong CO_2_ interactions with HCP restrict vibrational motion. At 150 °C, the broad HCP band at 1605 cm^–1^ originates from two narrower bands at 1605 and 1593 cm^–1^ (bands 3 and 4, Figure 3a). Both bands correspond to NH_2_ stretching vibrations coupled with C–NH_2_ and C–C vibrations, respectively. The positive bands at 1551 and 1490 cm^–1^ (bands 5 and 7, Figure 3b) become sharper and come from primary amine vibrations, specifically NH_2_ stretching vibrations coupled with C–NH_2_ and N–C=N vibrations, respectively. As all the positive bands which become sharper involve NH_2_ vibrations, we conclude that the primary amine groups of the triazine monomers are strongly involved in CO_2_ adsorption.

### 3.5 Vibrational Analysis during H_2_O Adsorption

We then investigated the HCP vibrational bands involved in H_2_O adsorption. For H_2_O adsorption measurements, we performed time-resolved DRIFTS measurements by alternatingly passing water saturated He and He through the DRIFT cell. We performed two H_2_O–He cycles for each temperature studied (50, 100, and 150 °C). Prior to passing water vapor through the DRIFTS cell, we collected a DRIFT spectrum of the HCP under He and used it as background spectrum to produce the difference spectra. To analyze our data, we applied the same approach as that followed for CO_2_ adsorption (see Section 3.4), and we describe our analysis in detail in the Supporting Information (Section S3). The analysis shows that at 50 °C H_2_O and CO_2_ adsorb in a similar manner on the HCP. However, we found that unlike CO_2_, H_2_O adsorbs more strongly to the biphenyl site than to the triazine one. This phenomenon likely results from strong hydrogen bonds between the water molecules and the intermolecular biphenyl C=O bonds.

### 3.6 Vibrational Band Analysis in the Presence of CO_2_ and H_2_O under Irradiation

**Figure 4:**
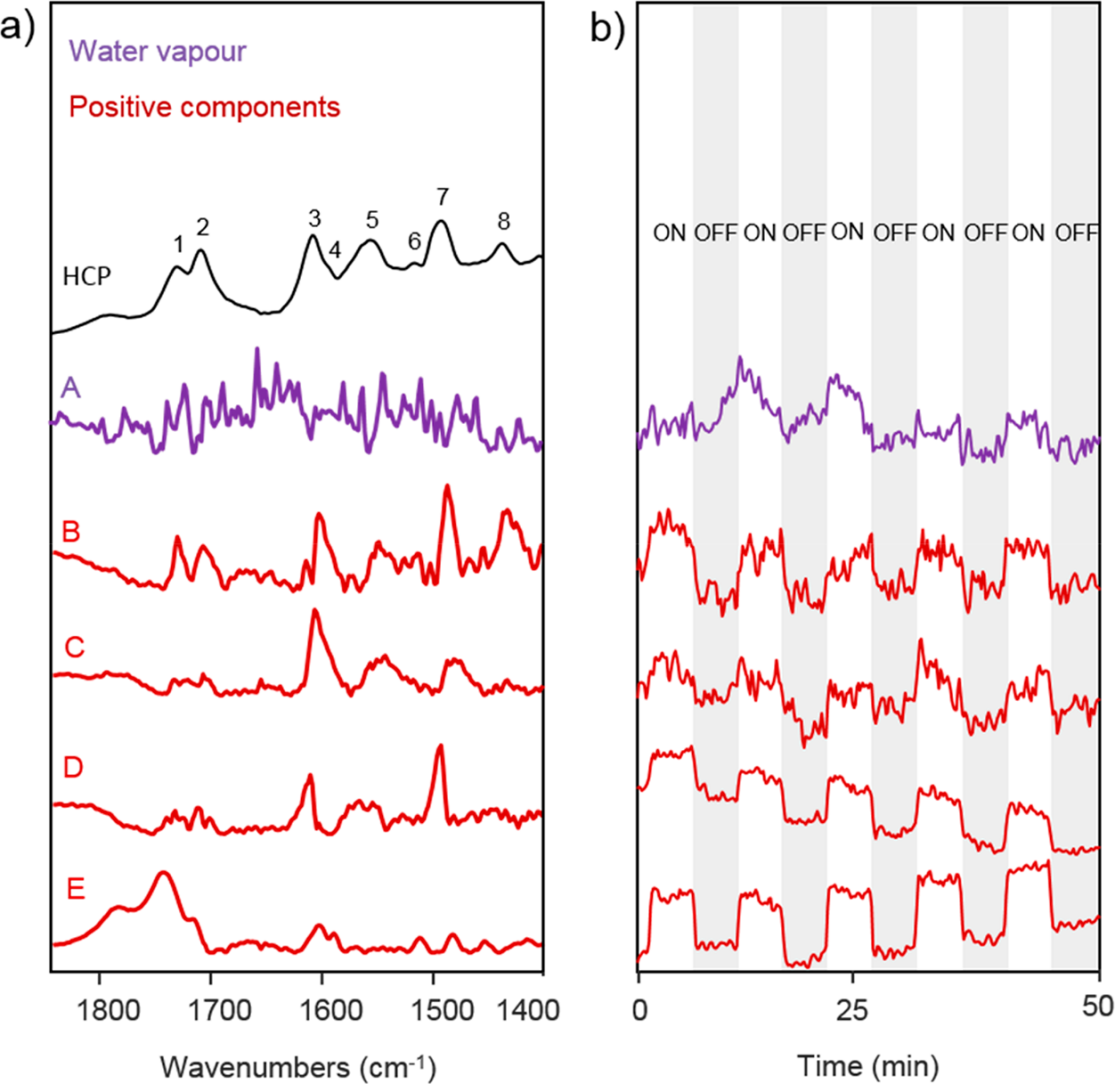
MCR components extracted from DRIFTS measurements during CO_2_ and H_2_O coadsorption on HCP under intermittent light irradiation. (a) Comparison between the DRIFT spectrum of the triazine–biphenyl HCP sample with that of water vapor (A) and the positive (B, C, D, E) spectral components obtained under a CO_2_/H_2_O atmosphere and transient UV–vis irradiation at 50 °C. (b) Component concentration profiles obtained by MCR.

Finally, we investigated CO_2_ and H_2_O coadsorption on HCP under transient light irradiation. During transient irradiation, we exposed the sample to a water-saturated CO_2_ atmosphere at 50 °C with UV–vis light turned on and off every 5 min. Prior to passing water-saturated CO_2_ through the DRIFTS cell, we collected a DRIFT spectrum of the HCP under He as background spectrum. We processed the difference spectrum obtained during the transient DRIFTS measurement using MCR. As Figure 4 shows, we extracted five different kinetically pure components with spectral significance. The first one (purple, component A) corresponds to water vapor present in the DRIFTS cell, while the others (components B–E) correspond to four different positive spectral components. As shown by the concentration profiles of the extracted components in Figure 4b, components B and C exhibit an amplitude change between light on and off similar to that of water vapor, while components D and E display more pronounced amplitude changes. Such signal difference likely arises from the different adsorption strengths between CO_2_ and H_2_O on the HCP. As the signal amplitude of components B and C is like that of water vapor, we assign these changes to water adsorption, while components D and E correspond to changes in CO_2_ adsorption. Compared to the CO_2_ components D and E, the smaller signal changes observed for H_2_O possibly arise from strong hydrogen bonds between the water molecules and the HCP, favoring strong interactions.

The concentration profile of the water vapor component suggests an increase of water vapor in the DRIFTS cell under light irradiation. This phenomenon likely results from water desorption caused by an increase of temperature at the HCP surface during irradiation. We also note that all the vibrational bands belonging to components B and C are red-shifted compared to that present in the background spectrum and dynamically appear and disappear under light on and off, respectively. Such behavior suggests an increase of substrate–HCP interactions upon light irradiation. Thus, despite partial H_2_O desorption, an increase of the number of interactions between the H_2_O molecules that are still adsorbed and the HCP occurs. As two components (B and C) can be extracted, we conject that the H_2_O molecules that are still adsorbed interact with the newly available adsorption sites in two different manners. The first one (component B) suggests that some of the adsorbed H_2_O molecules interact with both newly available biphenyl C=O bond (bands 1 and 2, Figure 4) and the triazine groups (bands 3 and 7), while the second component (component C) suggests that part of the adsorbed H_2_O only interacts with the newly available triazine groups as only bands 3 and 7 are visible.

**Figure 5:**
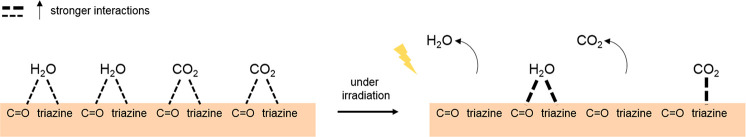
Schematic illustration of the impact of light irradiation upon CO_2_ and H_2_O interactions with the HCP. It highlights the changes in the number of interactions between the HCP and the substrates. We note that the latter is relative to the number of interactions detected under light off.

With regard to the CO_2_ components, the vibrational bands belonging to component E are blue-shifted compared to those in the background spectrum and dynamically appear and disappear under light on and off, respectively. As blue-shifted bands result from a decrease of substrate interactions and component E is mainly constituted of two vibrational bands corresponding to the biphenyl C=O bond vibrations (bands 1 and 2 of Figure 4), we suggest that under light irradiation adsorbed CO_2_, which originally interacts with both C=O and the triazine component, desorbs from the bridging C=O biphenyl site. In addition, the vibrational bands of component D, which correspond to the triazine group vibrations (bands 3 and 7, Figure 4) are red-shifted compared to the one present in the background spectrum and dynamically appear and disappear under light on and off, respectively, suggesting that upon light irradiation the number of CO_2_–triazine interactions increases. As under irradiation some H_2_O molecules desorb, partial CO_2_ desorption will also likely occur, generating newly available adsorption sites. Thus, we conject that upon light irradiation CO_2_ molecules that are still adsorbed will interact with the newly available triazine adsorption sites. Overall, upon light irradiation, we observe partial H_2_O and CO_2_ desorption followed by a redistribution of the number of interactions between the H_2_O and CO_2_ molecules that are still adsorbed and the HCP adsorption sites. We illustrate the impact of light irradiation upon CO_2_ and H_2_O interactions with the HCP in Figure 5.

Finally, we did not detect any intermediate species during the transient DRIFTS measurement, and we confirmed this by conducting a transient isotopic ^13^CO_2_ measurement and exposed the HCP to a water-saturated ^13^CO_2_ atmosphere instead of a water-saturated ^12^CO_2_ atmosphere and irradiated the sample with a UV–vis light 5 min intermittently. The analysis of the results is provided in the Supporting Information (Section S5, Figure S14). We note that isotopic experiments like this one, though rarely performed, remain important to unambiguously confirm the presence or not of intermediates.

## 4 Conclusion

We show a unique approach that combines DFT calculations, in situ DRIFTS, and MCR to probe gas (CO_2_) and vapor (H_2_O) interactions with carbon-based porous materials, demonstrated here on HCP. We did so at varying temperatures and with or without light irradiation. At 50 °C, CO_2_ adsorbs on both the triazine and biphenyl components of the HCP, albeit more strongly on the triazine one. The primary amine groups of triazine favored strong interactions with CO_2_. On the other hand, H_2_O adsorbed on the same sites but more strongly on the biphenyl component. Under transient light irradiation, we observed partial H_2_O and CO_2_ desorption and a redistribution of interactions between the still-adsorbed H_2_O and CO_2_ molecules and the HCP adsorption sites. Overall, this study helps gain a better understanding of the relationship between the HCPs structure/chemistry and CO_2_ and H_2_O adsorption. These findings can be used to develop improved HCP photocatalysts for CO_2_ conversion and better CO_2_ adsorbents.

## Supporting Information Available

Elemental composition, textural properties derived from N_2_ sorption isotherms, NMR, SEM, XPS spectra, pore size distribution, XRD, TGA, vibrational analysis and band assignment, proposed reaction synthesis of the triazine–biphenyl polymer, ^13^CO_2_ experimental data (PDF)
