# Supplementary material for: Evaluation of CO2 and H2O Adsorption on a Porous Polymer Using DFT and In Situ DRIFT Spectroscopy
Source: J Phys Chem B. 2022 Sep 28;126(40):8048–57. doi: 10.1021/acs.jpcb.2c03912 (PMC9574916; doi:10.1021/acs.jpcb.2c03912)
Supplement: Supplementary file 1 — jp2c03912_si_001.pdf [file jp2c03912_si_001.pdf]

Supporting Information

Evaluation of CO_2 and H_2O Adsorption on a Porous Polymer using DFT and in-situ DRIFT Spectroscopy

Giulia E. M. Schukraft[a] Ioanna Itskou,[a] Robert T. Woodward,[b] Bart Van Der Linden,[c] Camillle Petit[a*] and Atsushi Urakawa[c*]

[a]*Barrer Centre, Department of Chemical Engineering, South Kensington Campus, Imperial College London, London, SW7 2AZ, UK*

[b]*Institute of Materials Chemistry and Research, Faculty of Chemistry, University of Vienna, Währinger Straße 42, 1090 Vienna, Austria*

[c] *Catalysis Engineering, Department of Chemical Engineering, Delft University of Technology, Van der Maasweg 9, 2629 HZ Delft, The Netherlands*

*Corresponding authors: camille.petit@imperial.ac.uk; A.Urakawa@tudelft.nl

Section S1 – HCP characterization

Table S1. Elemental composition of the studied triazine-biphenyl HCP and the calculated atomic composition.

	C	N	O	H	S
w%	89.96	1.24	2.36	5.58	0.086
at%	56	0.62	1.1	42	0.02

The atomic composition was calculated using the following equation:

$$\text{C at\%} = \frac{\frac{\text{C w\%}}{\text{Cm}_a}}{\frac{\text{C w\%}}{\text{Cm}_a} + \frac{\text{Nw\%}}{\text{Nm}_a} + \frac{\text{Ow\%}}{\text{Om}_a} + \frac{\text{Hw\%}}{\text{Hm}_a} + \frac{\text{Sw\%}}{\text{Sm}_a}} \quad \text{(equation S1)}$$

where m_a represent the atomic mass of an element.

Table S2. Summary of textural properties derived from N_2 sorption isotherms at -196 °C, along with CO_2 adsorption capacities at 25 and 50 °C and 1 bar and H_2O adsorption capacities at 25 and 50 °C and 23 mbar for the studied HCP.

V_{MICRO} (cm^3/g)	V_{TOT} (cm^3/g)	S_{BET} (m^2/g)	CO_2 ads. at 25 °C (mmol/g)	CO_2 ads. at 50 °C (mmol/g)	H_2O ads. at 25 °C (mmol/g)	H_2O ads. at 50 °C (mmol/g)
0.136	0.978	1247	1.38	0.70	1.5	0.17

Table S3. CO_2 uptake capacity of different hypercrosslinked polymers reported in the literature.

Adsorbent	Surface area (m^2/g)	V_{Tot} (cm^3/g)	V_{micro} (cm^3/g)	CO_2 adsorption at 1 bar and 25 °C (mmol/g)	Ref
Xylene-biphenyl HCP	1970	-	-	1.7	[1]
Biphenyl HCP	1260	1.15	0.55	1.25	[2]
Benzene based HCP	950	1.07	0.46	1.25	[3]
Benzene based HCP	857	-	0.151	2.8	[4]
NH_2 functionalized HCP	77	-	-	0.63	[5]
Triazine-quinone based HCP	1130	1.02	-	1.40	[6]
Triazine-biphenyl HCP	1247	0.9789	0.14	1.38	This work

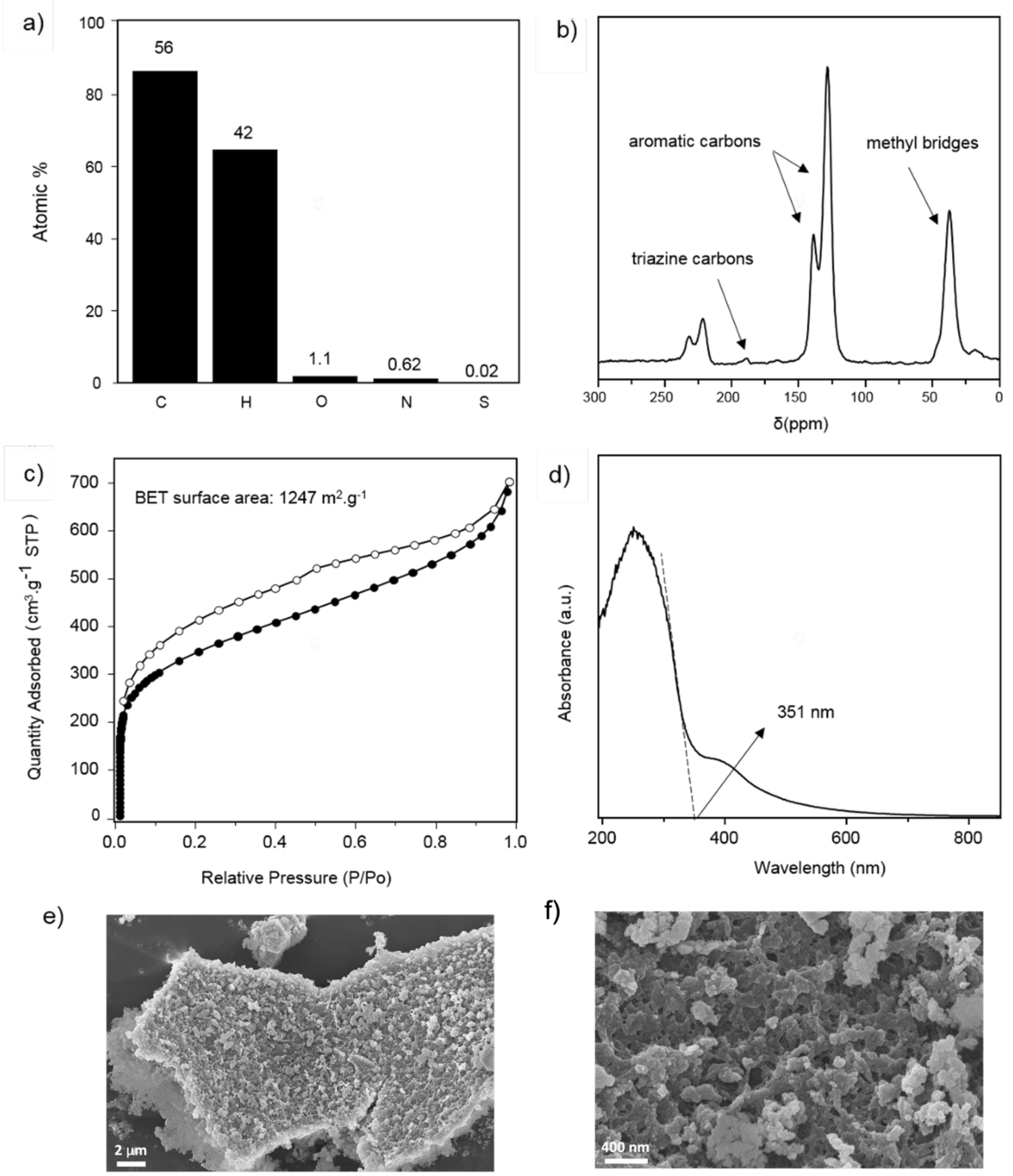

Figure S1. Overview of the chemical, structural, morphological and optoelectronic features of the triazine-biphenyl HCP sample: (a) atomic composition based on CHN-O-S analysis, (b) solid-state ^{13}C NMR spectra, (c) N_2 sorption isotherm at -196 °C (filled symbols = adsorption; empty symbols = desorption), (d) UV-vis absorption coefficient spectrum with absorption onset, (e, f) SEM images.

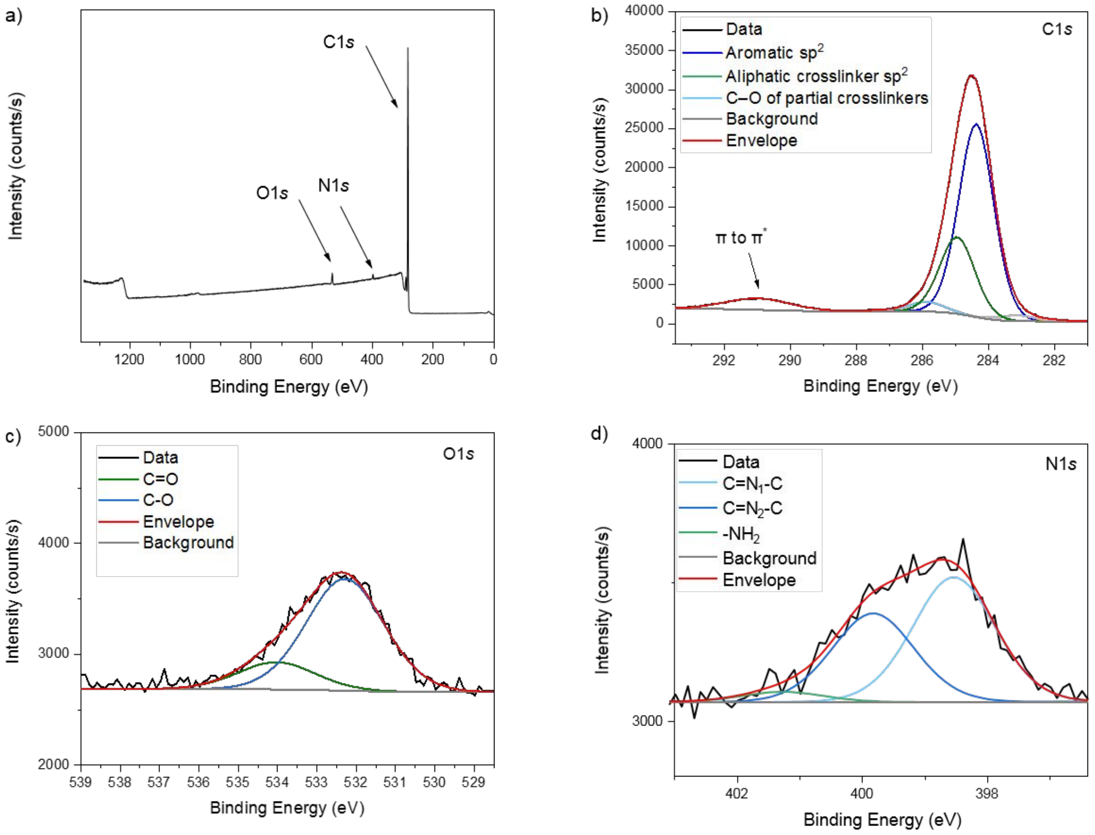

Figure S2. (a) XPS survey spectra, and high resolution XPS and peak fittings of the (b) C1*s*, c) O1*s* and d) N1*s* core levels of the HCP.

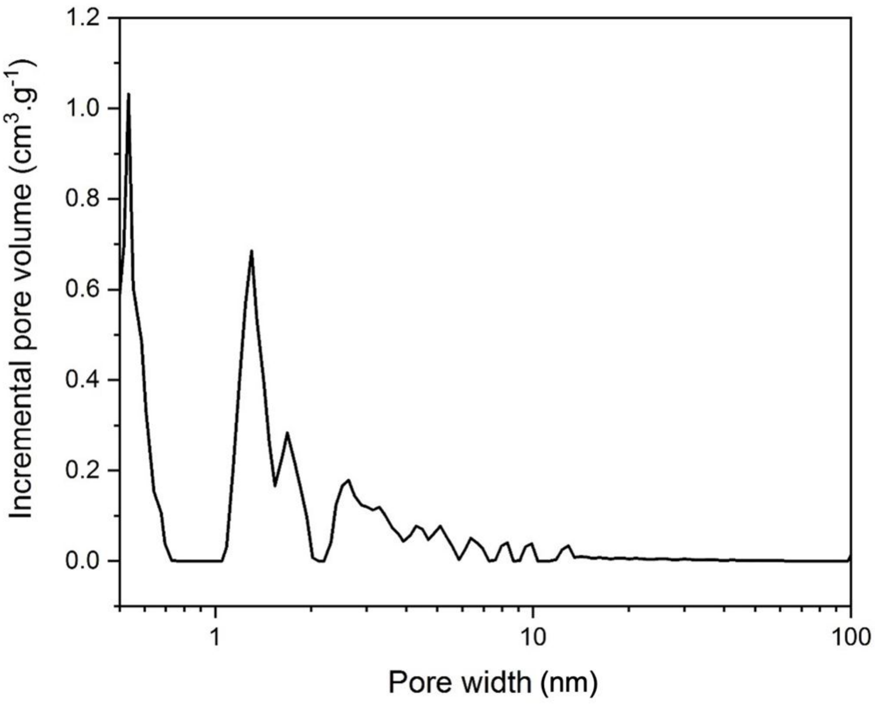

Figure S3. Pore size distribution of the triazine-biphenyl HCP.

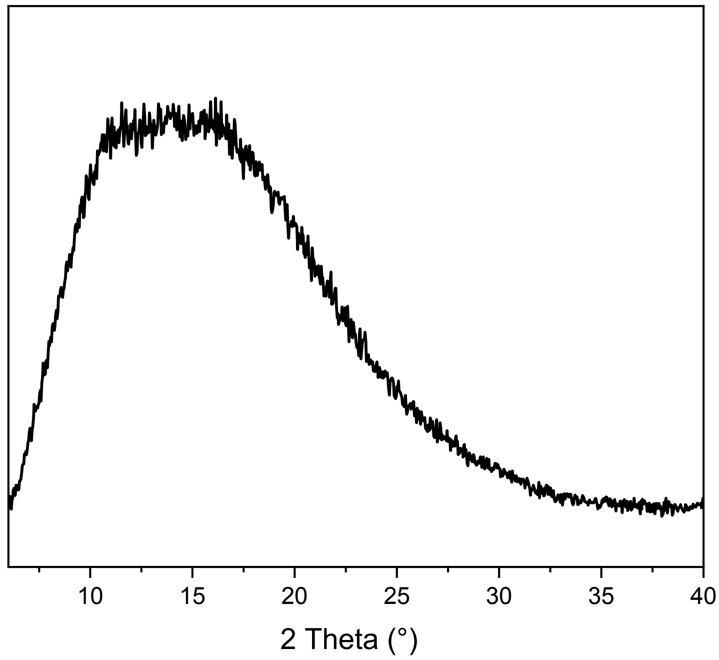

Figure S4. PXRD patterns of the triazine-biphenyl HCP.

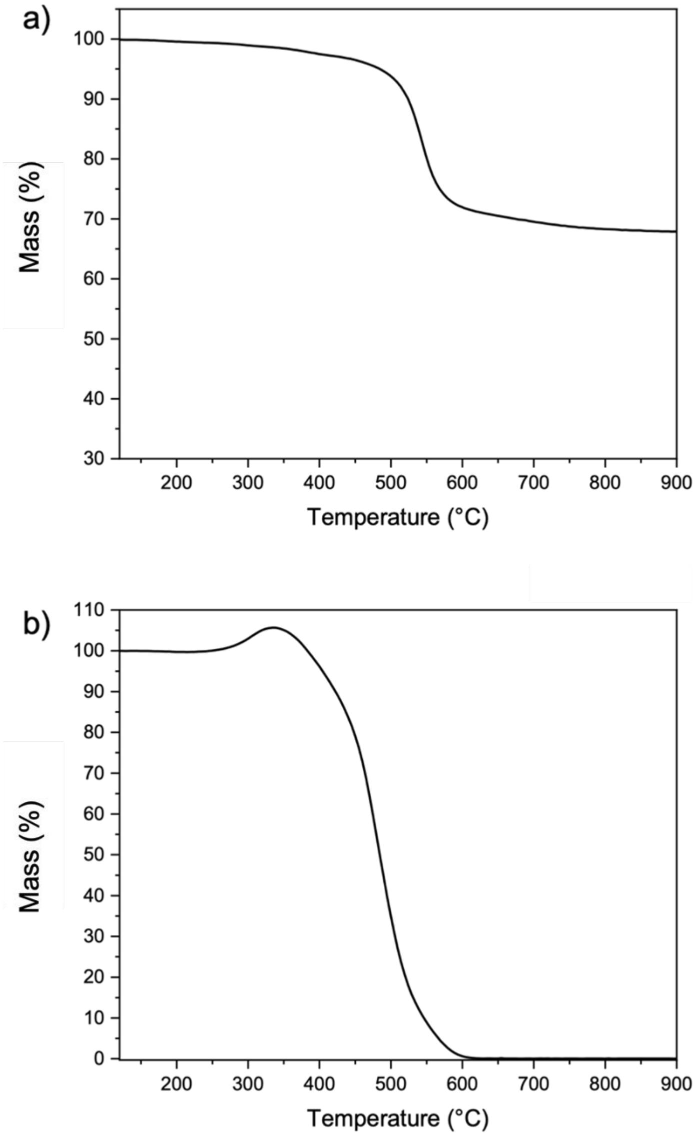

Figure S5. Thermogravimetric curves of the triazine-biphenyl HCP obtained under: (a) N_2 and (b) air.

Section S2 - Vibrational analysis and band assignment

To assign the triazine-biphenyl HCP vibrational bands one first needs to understand how intermolecular interactions impact the infrared spectrum of a triazine molecule and that of a biphenyl molecule. To do this, we evaluated the effect of triazine intermolecular interactions on its infrared spectrum by gradually increasing the number of triazine interactions in the computer simulations. The latter was carried out by computing the infrared spectra of one and six triazine molecules. Given the increasing computing cost, we studied the effect of more intermolecular interactions experimentally by collecting the ATR-IR spectrum of an 80 nM triazine solution and that of crystallized triazine powder. We focused the initial vibrational analysis on the spectral region ranging from 1800 to 1000 cm^{-1} where interactions with the substrate dominate. We discuss the vibrational analysis of other spectral regions below.

As Figure S6a and Table S4 show, triazine intermolecular interactions generate prominent spectral changes. An increase in intermolecular interactions, specifically in the number of their configurations, gives rise to band broadening. Among the measured spectra, the triazine powder infrared spectrum displaying the broadest band shapes resulting from an increased number of dipole-dipole interactions between the amino groups present in the triazine monomer. When increasing the number of intermolecular interactions, we also observe various band shifts. Bands 1, 2, and 5 of Figure S6a, which correspond to NH_2 stretching vibrations coupled with C-NH_2, C-C and C-N vibrations, according to the normal mode analysis (Table S4), respectively, gradually shifted towards higher wavenumbers. Additionally, we note the appearance of new vibrational bands (bands 3 and 4) that correspond to N-H vibrations coupled

with N-C=N vibrations. Overall, as Figures S6a shows, by gradually capturing the spectral implications of triazine intermolecular interactions, we can achieve a precise assignment of the triazine powder vibrational bands. As triazine powder is used as precursor for the HCP synthesis, the spectral features generated by strong intermolecular interaction will likely be present in the infrared spectrum of the HCP. A detailed assignment of the triazine powder vibrational bands can be found in Table S4.

To investigate the effect of the biphenyl intermolecular interactions on the IR spectral features, we followed a similar approach. The absence of strong polar groups in the biphenyl molecule and therefore, the lack of strong dipole-dipole intermolecular interactions, facilitated the assignment process. As Figure S6b shows, the simulated infrared spectrum of a single biphenyl molecule agrees well with the measured infrared spectrum of the biphenyl powder. The vibrational band at 1088 cm^{-1} (band 3, Figure S6b and Table S5) corresponds to the stretching vibration of the methoxy group. We observe two new vibrational bands at 1718 and 1697 cm^{-1} (bands 1 and 2, Figure S6b). As both vibrational bands do not correspond to any of the ones present in the computed infrared spectra of a single biphenyl molecules, they likely originate from biphenyl intermolecular interactions. The broad band tailing and their location at around 1700 cm^{-1} point to C=O bond vibrations. We assign these two vibrational bands to

the interaction of an aromatic carbon with an oxygen atom of a methoxy group present in another biphenyl molecule. A detailed assignment of the biphenyl powder vibrational bands can be found in Table S5.

The band assignment of the triazine and biphenyl powder, and the spectral implications of intramolecular interaction being confirmed, a precise analysis of the triazine-biphenyl HCP vibrational bands is now possible. As shown by Figure S6c, HCP exhibits characteristic vibrational bands of both triazine and biphenyl powder. Bands 1 and 2 of Figure S6c correspond to the vibrations of the intermolecular biphenyl C=O bond, while bands 3 to 9 correspond to the triazine component. Bands 3 and 4 correspond to NH_2 stretching vibrations coupled with C-N, and C-C ring vibrations. Lastly, band 10 corresponds to the vibration of unreacted methoxy groups arising from partially crosslinked biphenyl molecules. A more detailed assignment of the other HCP vibrational bands can be found in Table S6.

We note that all the aforementioned experimental infrared spectra were acquired using ATR-IR spectroscopy. As each infrared technique uses different sampling configurations, they will display different surface and bulk sensitivities and certain spectral features might differ.[7] To achieve a precise band assignment, one must compare spectra that are measured using identical IR techniques and necessary precautions needs to be taken when comparing spectra acquired using different sampling configurations, e.g., ATR-IR and DRIFTS. As Figure S7 shows, in the 1800 to 1000 cm^{-1} spectral region, the vibrational bands observed by ATR-IR and DRIFT spectra of the HCP are similar. We can therefore unequivocally transpose the obtained HCP band assignment based on the ATR-IR studies to that of the DRIFT spectra.

So far, we focused the vibrational analysis on the 1800 to 1000 cm^{-1} region. Yet, the 3650 to 3275 cm^{-1} region is also of interest as it corresponds to the 'pure' amino region (i.e. not coupled with aromatic vibrations). Interestingly, as Figure S6d shows, the measured ATR-IR spectrum

of the triazine-biphenyl HCP displays little amine vibrational bands. However, when performing DRIFTS analysis, reported below, we observe vibrational bands at 3530 and 3415 cm^{-1}, corresponding to the primary NH_2 asymmetric and symmetric stretching vibrations, respectively.[8] As mentioned above, this variance likely arises from the difference in measurement technique used. In ATR-IRS, the penetration depth of the infrared light depends on the incoming wavelength. At smaller wavelength, the penetration depth decreases, resulting in a loss of sensitivity at higher wavenumbers. Such phenomenon could explain the apparent absence of the primary amine bands at 3530 and 3415 cm^{-1}.

Though tangentially relevant to the objective of the study, we note that DRIFTS provided insights into the reaction mechanism linked to HCP formation. In brief, it supports the S_N1 route speculated in earlier studies, as shown in Figure S8.[2, 9] Upon protonation by the acid catalyst, a methoxy group of the biphenyl monomer leaves as methanol, forming a carbocation. The carbocation then undergoes nucleophilic attack from the triazine monomer and finally regeneration of the acid catalyst occurs by proton transfer. As we detected primary amine groups and no secondary amines, we conject that the phenyl ring of the triazine monomer, and not the primary amines, act as nucleophile. Overall, the stretching vibration of the primary amines observed using DRIFTS unveiled the synthesis mechanism of the triazine-biphenyl HCP.

Table S4. Comparison between the experimental ATR infrared spectrum of triazine powder and the simulated infrared spectrum of one triazine molecule, enabling assignment of the triazine monomer vibrational bands. To allow comparison between the simulated and the measured infrared spectra, a scaling factor of 0.973 was applied to the simulated one.

Band number as labeled in Figure S6a	Experimental vibrational frequency of triazine powder	Calculated vibrational frequency	Assignment	Graphical illustration of the displacement vector

	(cm^{-1})	$(cm^{-1})^{*}$		
1	1616	1615	s(NH_2) + v(C-NH$_2$)	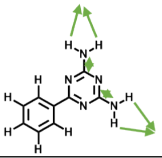
2	1603	1609	s(NH_2) + v(C-C)	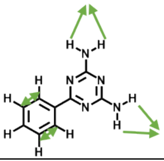
3	1590	1603	s(NH_2) +N-C=N + C-C=C	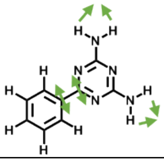
4	1557	1563	v(NH) +N-C=N	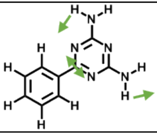
5	1531	1557	v(C-N) + v(NH)	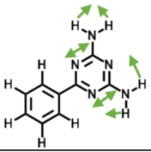
6	1506	1493	s(NH_2) + v(C-NH$_2$) + benzene ring vibrations	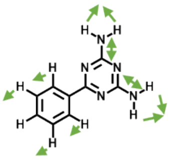
7	1487	1472	s(NH_2) + v(C-NH$_2$) + benzene ring vibrations	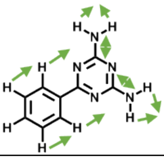
8	1450	1450	s(NH_2) + benzene ring vibrations	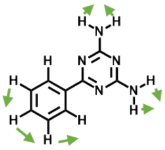
9	1425	1421	s(NH_2) + v(C-NH$_2$) + benzene ring vibrations	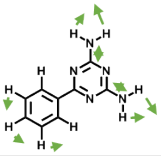
-	1387	1394	v(C-C) between benzene and triazine ring	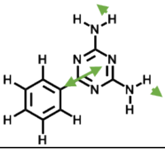

-	1257	1264	v(C-N) + v(NH)	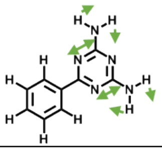
-	1192	1153	benzene ring vibrations	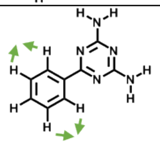

*Based on the simulated infrared spectra of 1 triazine molecule.

Definition: δ: bending vibration; ν: stretching vibration; s: scissoring.

Table S5. Comparison between the experimental ATR infrared spectra of biphenyl powder and the simulated infrared spectra of one biphenyl molecule, enabling assignment of the biphenyl monomer vibrational bands. To allow comparison between the simulated and the measured infrared spectra a scaling factor of 0.975 was applied to the simulated one.

Band number as labeled in Figure S6b	Experimental vibrational frequency of biphenyl powder (cm^{-1})	Calculated vibrational frequency (cm^{-1})	Assignment	Graphical illustration of the displacement vector
1<br>2	1718<br>1697	-	intermolecular C=O vibrations	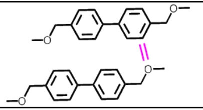
3	1602	1623	ν(C-C) of the benzene ring	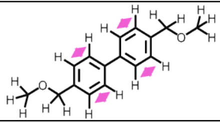
4	1579	1586	ν(C-C) of the benzene ring	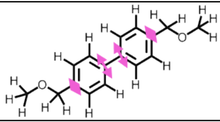
5	1561	1566	ν(C-C) of the benzene ring	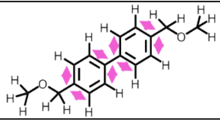
6	1498	1502	benzene ring vibrations	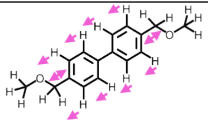
7	1450	1446	benzene ring vibrations	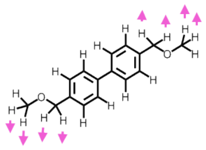
8	1399	1399	ν(C-C) of the benzene ring	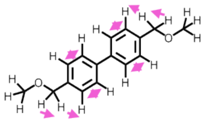
9	1380	1371	δ(CH_2) of the methylene group	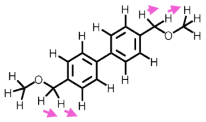
10	1364	1369	benzene ring vibrations	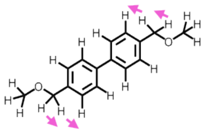
11	1312	1305	benzene ring vibrations	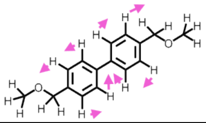

12	1275	1275	ν(C-C)	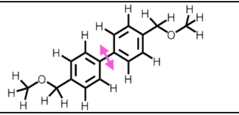
13	1208<br>1190	1208<br>1189	δ(CH_2) of the methylene group + δ(CH_3) of the methoxy group	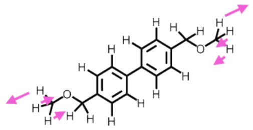
14	1088	1137	ν(C-O)	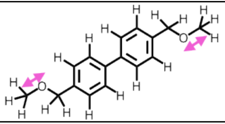

*Based on the simulated infrared spectra of 1 biphenyl molecule

Definition: δ: bending vibration; ν: stretching vibration; s: scissoring.

Table S6. Vibrational analysis and assignment of the triazine-biphenyl HCP.

Band number as labeled in Figure S6c	Experimental vibrational frequency (cm^{-1})	Monomer contribution	Assignment	Graphical illustration of the displacement vector
1<br>2	1726<br>1704	biphenyl	Intermolecular C=O vibrations	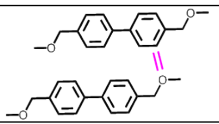
3	1605	triazine	s(NH_2) + v(C-NH_2)	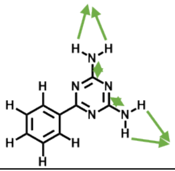
4	1593	triazine	s(NH_2) + v(C-C) benzene ring	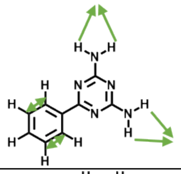
5	1551	triazine	v(N-H) + N-C=N stretching	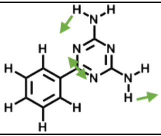
6	1515	triazine	v(C-N) + v(NH)	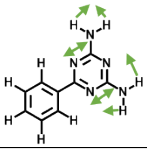
7	1490	triazine	s(NH_2) + v(C-NH_2) + benzene ring vibrations	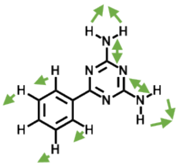
8	1457	triazine	s(NH_2) + v(C-NH_2) + benzene ring vibrations	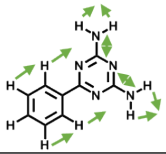
9	1433	triazine	s(NH_2) + benzene ring vibrations	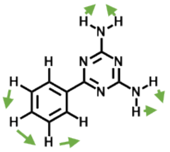
-	1400	biphenyl	v(C-C) of the benzene ring	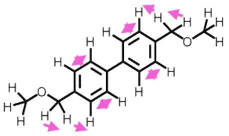
-	1390	biphenyl	$\delta(CH_2)$ of the methylene group	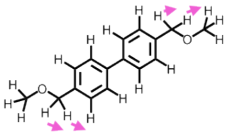

-	1332	biphenyl	benzene ring vibrations	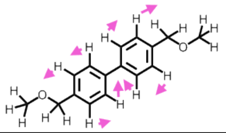
-	1246	biphenyl	ν(C-C)	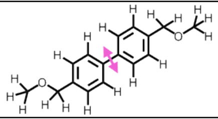
-	1238<br>1211<br>1182	biphenyl	δ(CH_2) of the methylene group + δ(CH_3) of the methoxy group	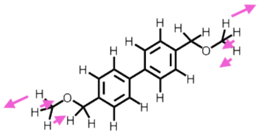
10	1110	biphenyl	ν(C-O)	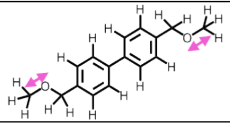

Definition: δ: bending vibration; ν: stretching vibration; s: scissoring.

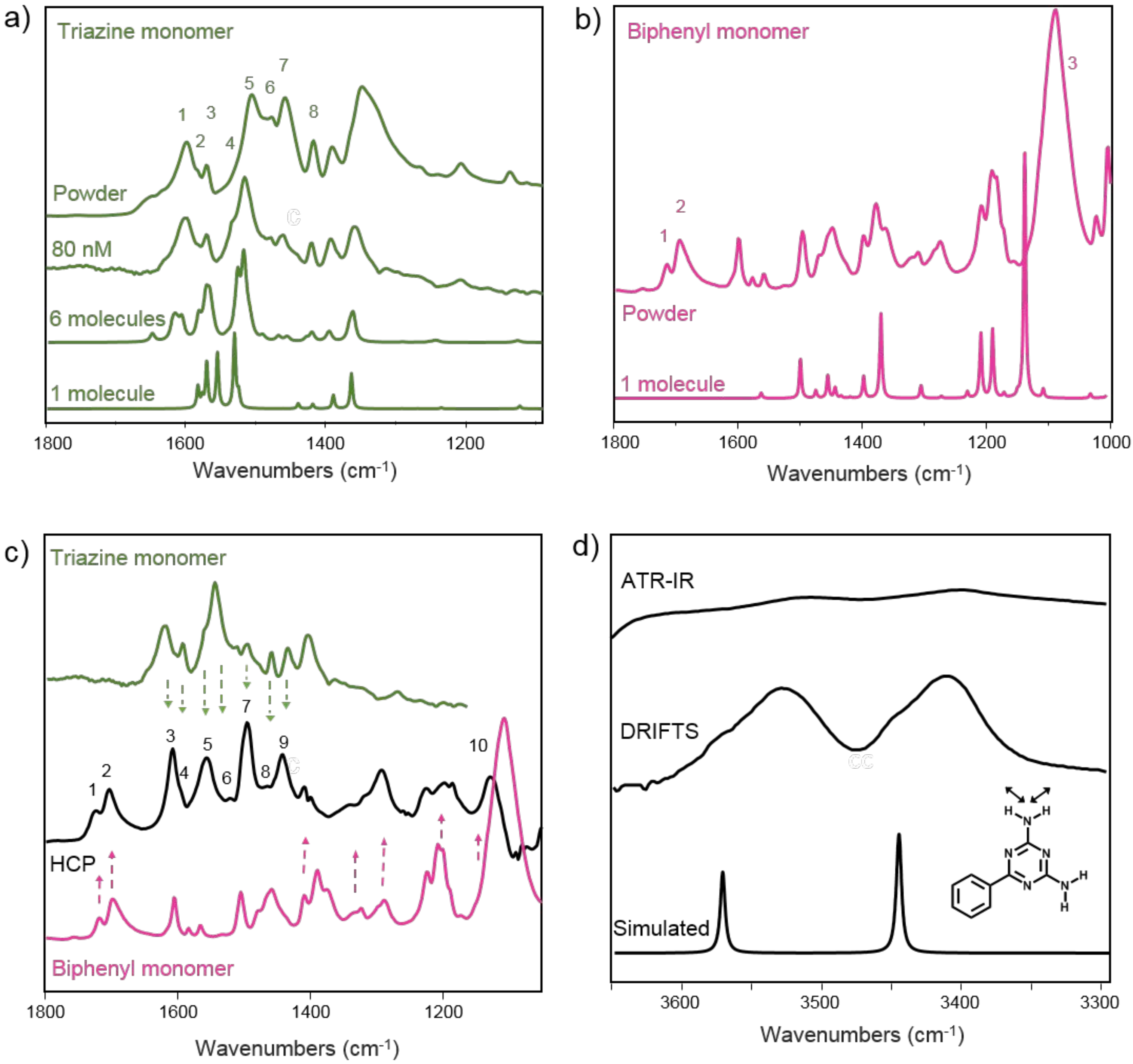

Figure S6. Vibrational analysis and band assignment for the triazine-biphenyl HCP sample: (a) simulated infrared spectra of one and six triazine molecules, experimental ATR infrared spectrum of an 80 nM triazine monomer solution and triazine monomer powder. (b) Simulated infrared spectrum of one biphenyl monomer molecule and experimental ATR infrared spectrum of the biphenyl monomer powder. (c) Comparison between the ATR infrared spectra of the triazine and biphenyl monomer with that of the triazine-biphenyl HCP. (d) Comparison between the ATR-IR and DRIFT spectra of the HCP and the simulated one.

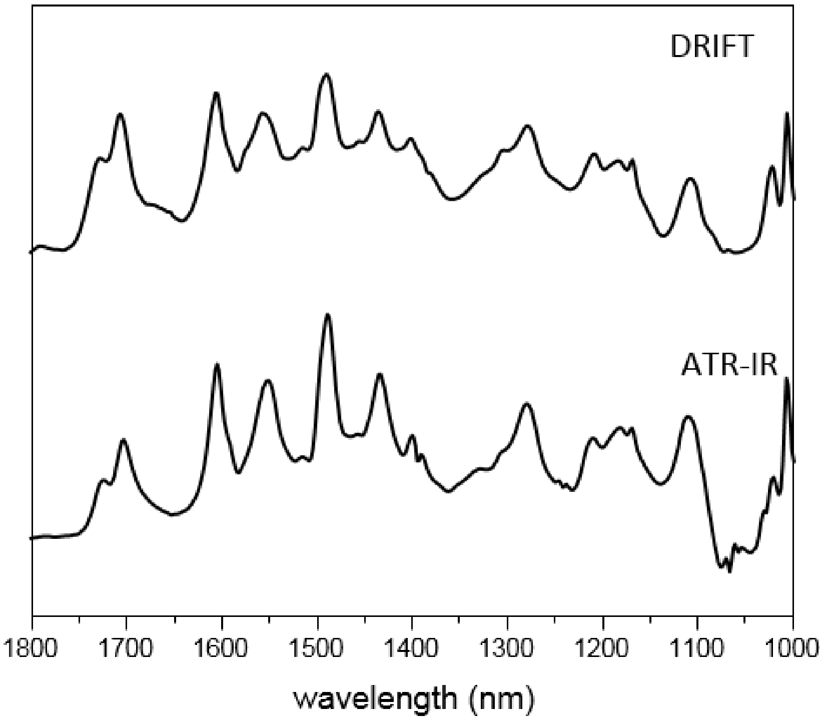

Figure S7. Comparison of the DRIFT and ATR-IR spectra of the triazine-biphenyl HCP.

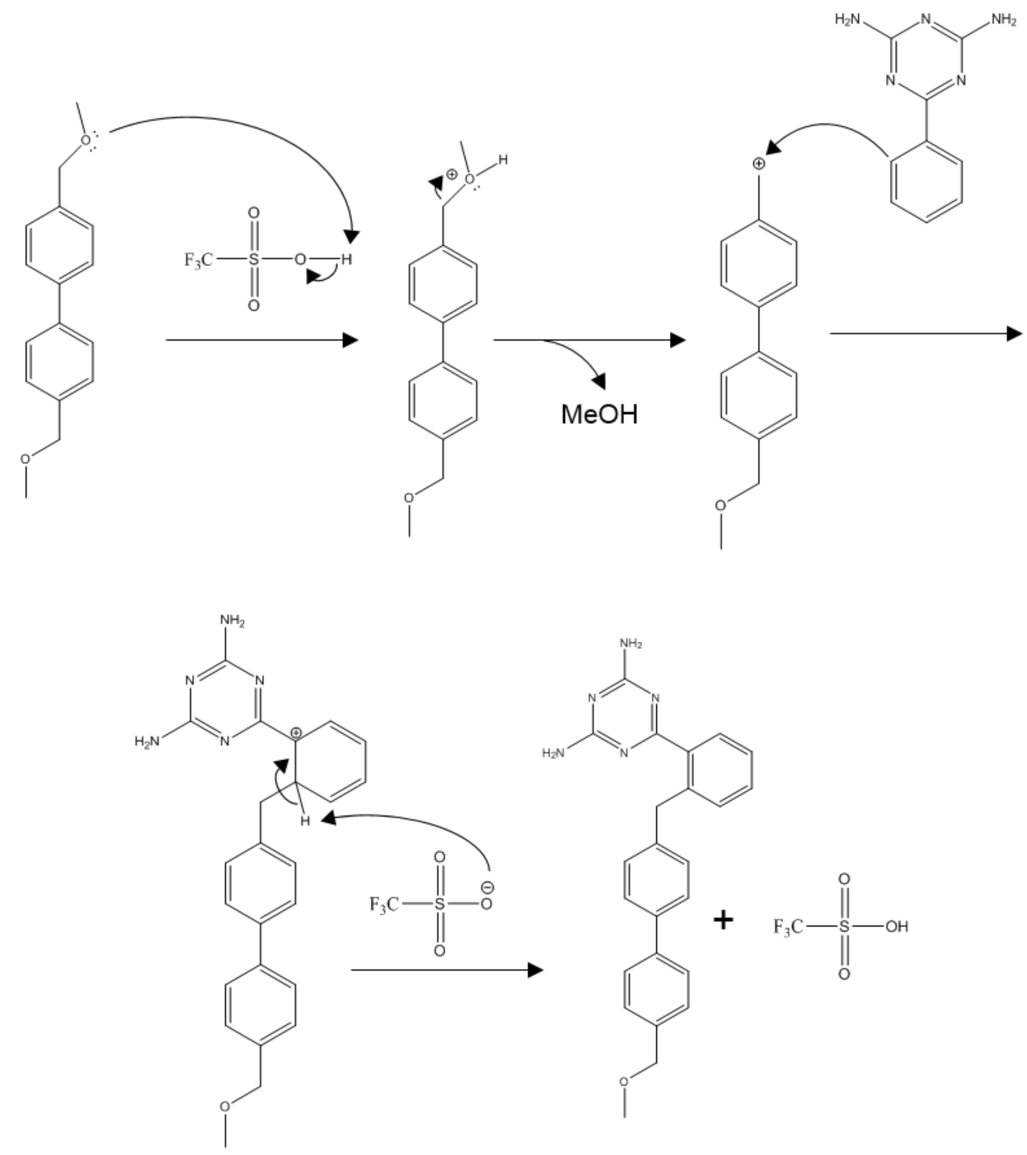

Figure S8. Proposed reaction synthesis of the triazine-biphenyl HCP when using triflic acid as polymerization catalyst.

Section S3 - Vibrational band analysis during CO_2 adsorption

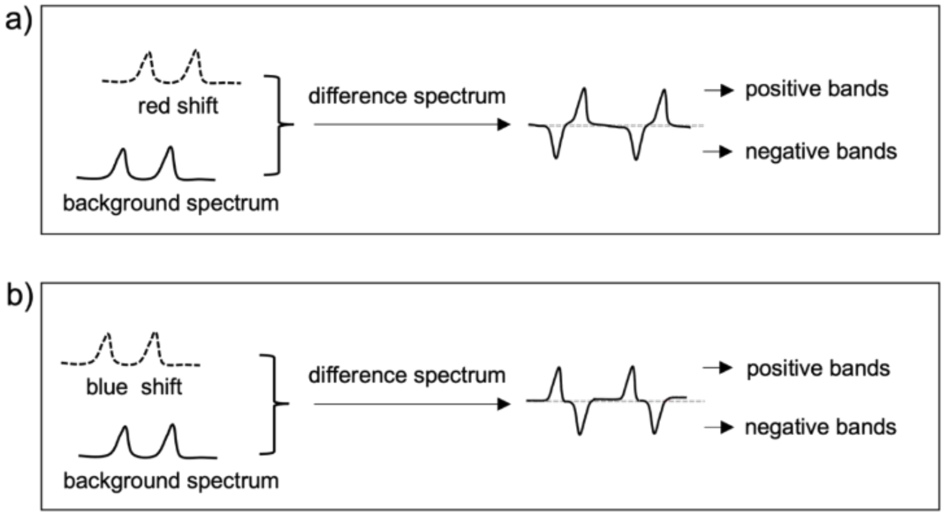

Figure S9. Representation of the origin of positive and negative bands in a difference spectrum. Panel (a) illustrates the type of difference spectrum obtained upon a red shift of the vibrational bands of a material compared to its background spectrum. Panel (b) illustrates the type of difference spectrum obtained upon a blue shift of the vibrational bands of a material compared to its background spectrum.

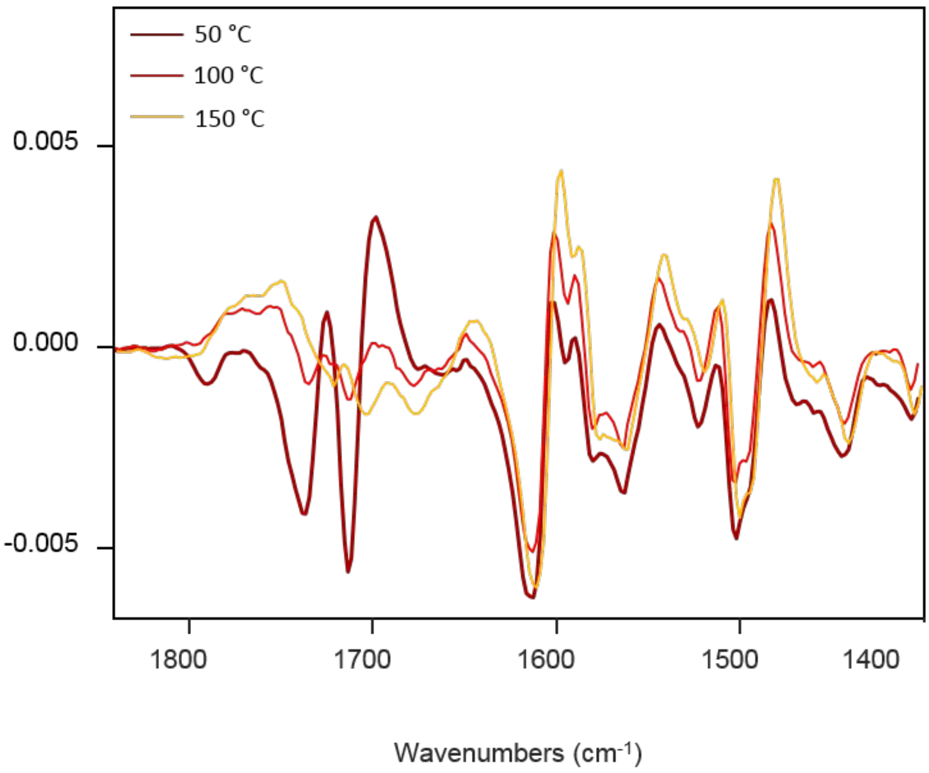

Figure S10. Average of the difference spectra obtained under a CO_2 atmosphere at 50, 100, 150 °C.

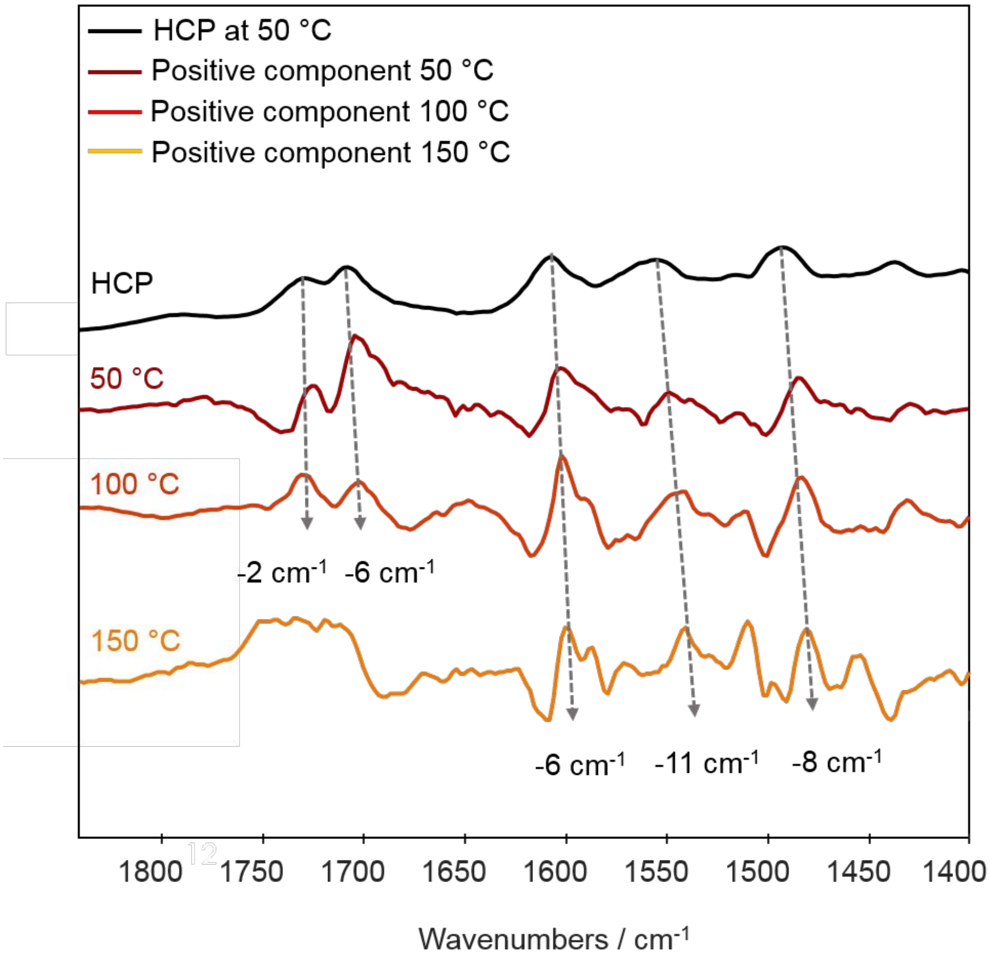

Figure S11. Comparison between the vibrational band positions of the positive components extracted by MCR at 50, 100 and 150 °C and the corresponding unshifted HCP vibrational bands.

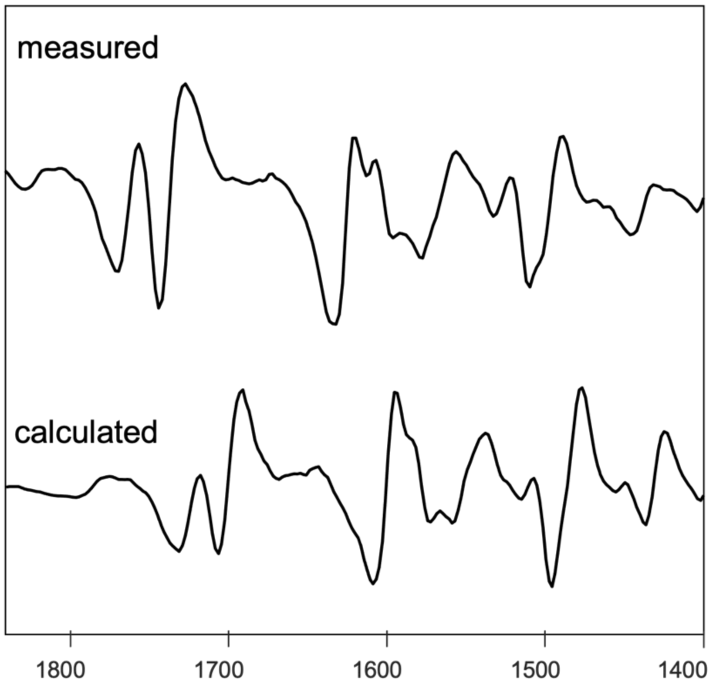

Figure S12. Comparison between the measured difference spectrum under a CO_2 atmosphere at 50 °C and the calculated one. The calculated difference spectrum is obtained by mathematically shifting the background spectrum by -8 cm^{-1}, and the latter is subtracted to the original background spectra.

Section S4 - Vibrational band analysis during H_2O adsorption

As Figure S13a shows, in the presence of H_2O at 50 ˚C, the difference spectrum displays negative and positive bands, suggesting that some of the HCP vibrational bands are shifted due to interactions between HCP and H_2O. Following the same reasoning as for CO_2 adsorption, as negative bands precede positive ones, we infer that some of the HCP vibrational bands are red shifted. We then processed the time-resolved spectra using MCR to determine which HCP vibrational bands shifted upon interactions with H_2O. As water vapor absorbs infrared light, it creates an undesirable background noise in the 2000-1300 cm^{-1} region and one should separate this effect from that of water adsorption in HCP. Thanks to the different kinetic behavior of water vapor and that of adsorbed water, MCR can resolve the water vapor contribution and separate it from the other spectral components (Figure S13a).

As Figure S13a shows, when processing the difference spectrum obtained under a H_2O atmosphere using MCR, we extract three spectrally significant and kinetically pure components. The first component (purple, Figure S13b) corresponds to the water vapor present in the DRIFTS cell, the second component (red, Figure S13c) to the positive spectral features of the difference spectrum, and the third component (blue, Figure S13d) to the negative spectral features of the difference spectrum. As shown in Figure S13c, at 50 ˚C, the positive component

displays vibrational bands at around 1726, 1704, 1605, 1551 and 1490 cm^{-1} (bands 1, 2, 3, and 7) and the negative component display vibrational bands at around 1726, and 1704 cm^{-1} (bands 1 and 2 of Figure S13d). The HCP vibrational bands located at those wavenumbers are the ones shifted upon H_2O adsorption. Interestingly, the shifted HCP vibrational bands in presence of H_2O are identical to those shifted in presence of CO_2. At 50 ˚C, as for CO_2, H_2O interacts with the intermolecular C=O bond of the biphenyl component, the primary amines and the triazine groups of the triazine monomer. Additionally, all the positive vibrational bands belong to the same kinetically pure components and therefore dynamically appear or disappear at the same time in presence or absence of H_2O. A similar reasoning can be made for the negative vibrational bands. As for CO_2 adsorption, these observations indicate that the HCPs vibrational bands located at those wavenumbers are all shifting at the same rate in the presence of H_2O and suggest that H_2O interacts simultaneously with all five functional groups. In short, at 50 ˚C, H_2O and CO_2 adsorb in a similar manner on the HCP.

To investigate the strength of H_2O adsorption, we conducted additional H_2O adsorption measurements at 100 ˚C and 150 ˚C. As Figure S13c and d show, at 100 ˚C, the positive vibrational bands linked to the triazine monomer (bands 3 and 7 and of Figure S13c and d), decrease faster than those of the intermolecular biphenyl C=O bond (bands 1 and 2). Hence,

unlike CO_2, H_2O adsorbs more strongly to the biphenyl site than to the triazine one (Figure S13e). This phenomenon likely results from strong hydrogen bonds between the water molecules and the intermolecular biphenyl C=O bonds. At 150 ˚C, due to very low H_2O adsorption, we did not extract meaningful data.

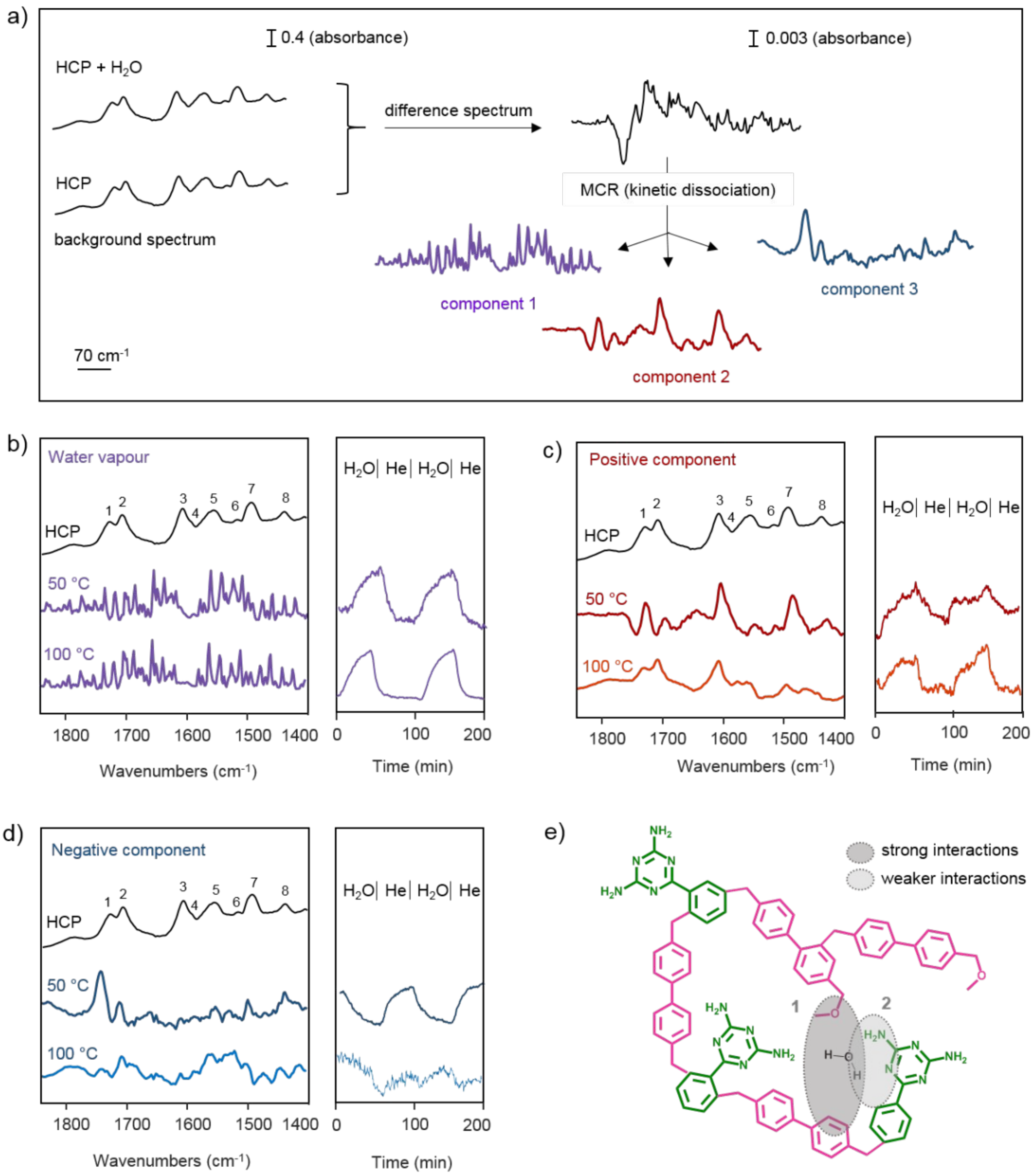

Figure S13. DRIFTS study of H_2O adsorption on HCP. (a) Schematic illustration of the spectral components obtained after processing the difference spectrum using MCR. The

difference spectrum corresponds to the average of the difference spectrum acquired under a H_2O atmosphere at 50 °C. Left panels of (b), (c) and (d): comparison between the DRIFT spectrum of the triazine-biphenyl HCP sample with: water vapor (b), the positive (c) and negative (d) spectral components obtained for H_2O adsorption measurements at 50 °C and 100 °C after performing MCR analysis. Right panels of (b), (c) and (d): component concentration profiles obtained by MCR. (e) Illustration of the H_2O adsorption sites in HCP.

Section S5 - Vibrational band analysis in the presence of CO_2 and H_2O under irradiation

Isotopic study to study intermediates: Compared to $^{12}CO_2$, the vibrational bands of $^{13}CO_2$ should be red-shifted. As Figure S14a shows, under irradiation, when switching from a $^{12}CO_2/H_2O$ to a $^{13}CO_2/H_2O$ atmosphere, the spectroscopic signature of CO_2 shifted. Such shift is clearly visible for the combination bands located at 3725, 3700, 3627, and 3600 cm^{-1}, and the symmetric and asymmetric CO_2 stretching bands located at ~2350 and 2330 cm^{-1}, respectively. Yet, in the 2000 - 1400 cm^{-1} spectral region, where we expect the vibrational bands of intermediate species such as carboxylate, the difference spectra obtained under a $^{13}CO_2/H_2O$ atmosphere resembles that obtained under a $^{12}CO_2/H_2O$ atmosphere (Figure S14b). These observations suggest that no intermediate species are observed under irradiation. At an early stage of our transient DRIFTS data analysis, we assigned component E of Figure 5 to be a carboxylate species due to its bands' locations at around 1750 cm^{-1}. Thanks to the isotopic measurement, we can conclude that component E is not an intermediate species.

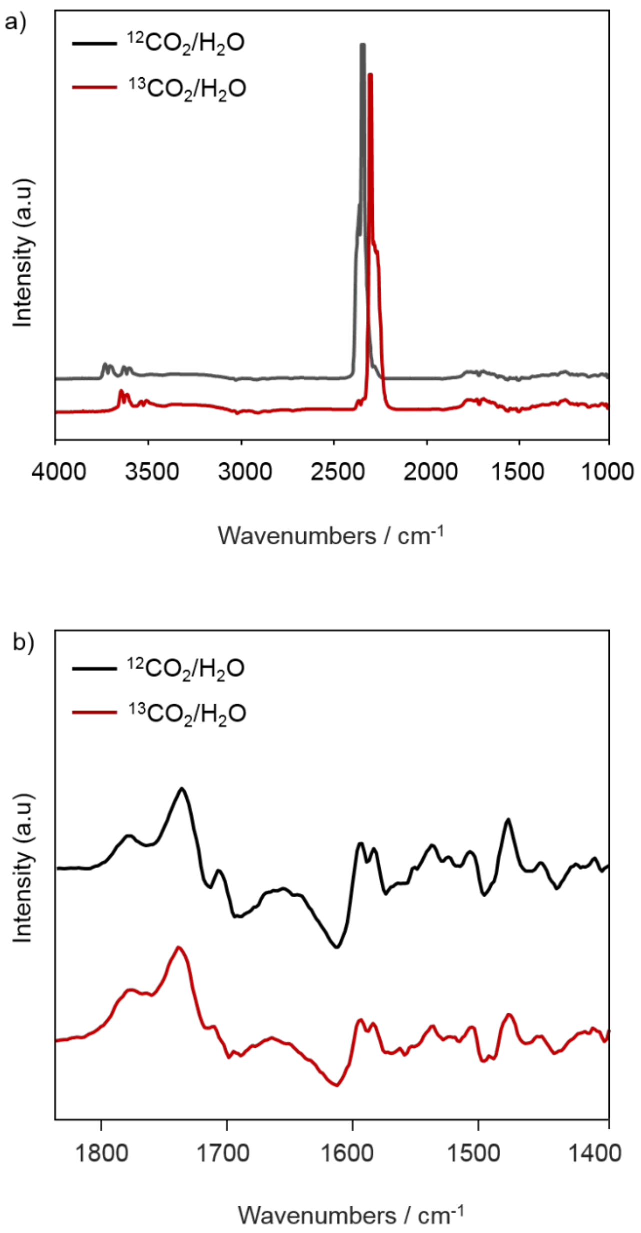

Figure S14. Comparison between the difference spectra obtained under light irradiation under a $^{12}CO_2/H_2O$ and a $^{13}CO_2/H_2O$ in the (a) 4000-1000 cm^{-1} and (b) 1840 -1400 cm^{-1} regions.

References

1. Martin, C. F.; Stockel, E.; Clowes, R.; Adams, D. J.; Cooper, A. I.; Pis, J. J.; Rubiera, F.; Pevida, C., Hypercrosslinked organic polymer networks as potential adsorbents for pre-combustion CO2 capture. *J Mater Chem* **2011,** *21* (14), 5475-5483.
2. Woodward, R. T., The design of hypercrosslinked polymers from benzyl ether self-condensing compounds and external crosslinkers. *Chem Commun* **2020,** *56* (36), 4938-4941.
3. Schukraft, G. E. M.; Woodward, R. T.; Kumar, S.; Sachs, M.; Eslava, S.; Petit, C., Hypercrosslinked Polymers as a Photocatalytic Platform for Visible-Light-Driven CO2 Photoreduction Using H2O. *Chemsuschem* **2021,** *14* (7), 1720-1727.
4. Ramezanipour Penchah, H.; Ghanadzadeh Gilani, H.; Ghaemi, A., CO2, N2, and H2 Adsorption by Hyper-Cross-Linked Polymers and Their Selectivity Evaluation by Gas–Solid Equilibrium. *J Chem Eng Data* **2020,** *65* (10), 4905-4913.
5. Su, P.; Zhang, X.; Xu, Z.; Zhang, G.; Shen, C.; Meng, Q., Amino-functionalized hypercrosslinked polymers for highly selective anionic dye removal and CO2/N2 separation. *New J Chem* **2019,** *43* (44), 17267-17274.
6. Mohamed, M. G.; Zhang, X.; Mansoure, T. H.; El-Mahdy, A. F. M.; Huang, C. F.; Danko, M.; Xin, Z.; Kuo, S. W., Hypercrosslinked porous organic polymers based on tetraphenylanthraquinone for CO2 uptake and high-performance supercapacitor. *Polymer* **2020,** *205*, 122857.
7. Roedel, E.; Urakawa, A.; Kureti, S.; Baiker, A., On the local sensitivity of different IR techniques: Ba species relevant in NOx storage-reduction. *Phys Chem Chem Phys* **2008,** *10* (40), 6190-6198.
8. Yuan, X. H.; Luo, K.; Zhang, K. Q.; He, J. L.; Zhao, Y. C.; Yu, D. L., Combinatorial Vibration-Mode Assignment for the FTIR Spectrum of Crystalline Melamine: A Strategic Approach toward Theoretical IR Vibrational Calculations of Triazine-Based Compounds. *J Phys Chem A* **2016,** *120* (38), 7427-7433.
9. Zafar, M. S.; Liaqat, S.; Najeeb, S.; Khurshid, Z.; Alrahabi, M.; Zohaib, S. In *Polymer science : research advances , practical applications and educational aspects*, 2015.
